# A high-resolution in situ X-ray diffraction study of mineral transitions due to post-hydration heating in CM chondrite meteorites

**DOI:** 10.1186/s40623-024-02116-2

**Published:** 2024-12-26

**Authors:** Laura E. Jenkins, Ashley J. King, Martin R. Lee, Luke Daly, Stephen P. Thompson, Sarah J. Day, Lucy Saunders, Pierre-Etienne Martin, Fahkri Bintang

**Affiliations:** 1 School of Geographical and Earth Sciences, The University of Glasgow, Glasgow, G12 8RZ Scotland, UK; 2 Planetary Materials Group, Natural History Museum, London, SW7 5BD England, UK; 3 Space Science and Technology Centre, School of Earth and Planetary Sciences, Curtin University, Perth, 6845 Australia; 4 Australian Centre for Microscopy and Microanalysis, The University of Sydney, Sydney, 2006 Australia; 5 Present Address: Diamond Light Source, Harwell Science and Innovation Campus, Didcot, Oxfordshire OX11 0DE England, UK

**Keywords:** Carbonaceous chondrites, Meteorites, Thermal metamorphism, Post-hydration heating, X-ray diffraction, Laboratory experiments

## Abstract

**Abstract:**

The effects of post-hydration heating over a broad range of temperatures are evident in many Mighei-like carbonaceous (CM) chondrites as a variety of mineral transitions. To better understand these processes and how a CM chondrite’s starting composition may have affected them, we experimentally heated two meteorites with different degrees of aqueous alteration, Allan Hills 83100 and Murchison, at 25 °C temperature steps from 200 °C to 950 °C and 300 °C to 750 °C, respectively. During heating, synchrotron in situ X-ray diffraction patterns were collected. With the exception of calcite decomposition and its products, most mineral transitions were unaffected by starting composition. Key observations include: (1) partial decomposition of tochilinite at 200 °C, which indicates that tochilinite breakdown might be a two-stage process due to its intergrown layers of brucite/amakinite and mackinawite; (2) the breakdown of serpentine occurring at 300 °C with transitional phases appearing at 525 °C and 575–600 °C, while secondary olivine formed at 600 °C; (3) cronstedtite decomposing faster than lizardite, (4) the formation of secondary enstatite at 750 °C, and (5) calcite decomposition temperature differing significantly between meteorites, occurring at 725 °C and 575 °C in ALH 83100 and Murchison, respectively. The results for calcite are likely controlled by differences in its microstructure and chemical composition, related to the meteorite’s impact history and degree of aqueous alteration. The difference in calcite decomposition temperature also explains the contrasts in the observed breakdown products, with clinopyroxene occurring in both meteorites, and oldhamite only in ALH 83100. Mineral transitions due to post-hydration heating have been characterized with a high resolution XRD method, enabling a better understanding of processes occurring on the parent asteroids of CM chondrites.

**Graphical Abstract:**

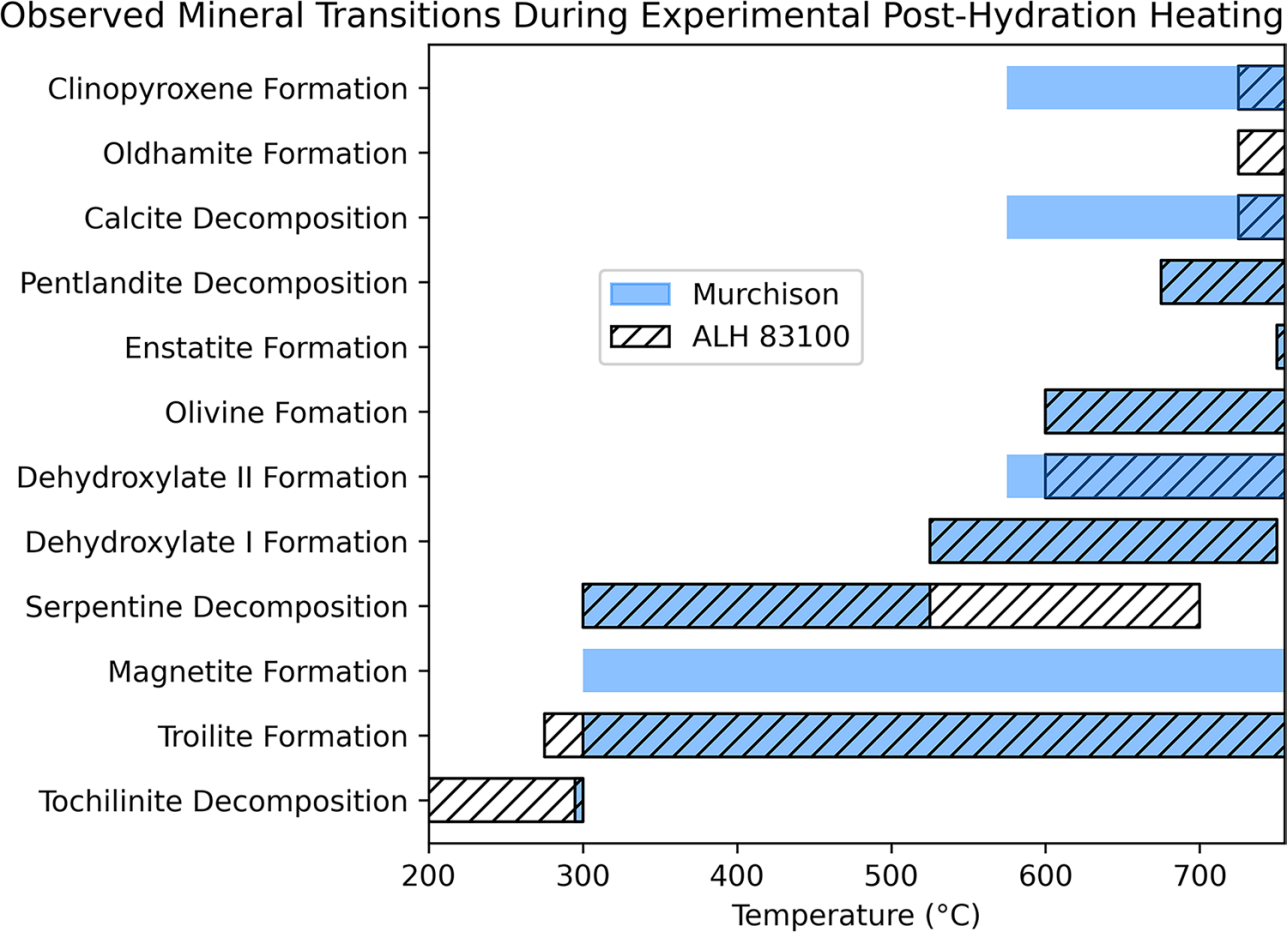

**Supplementary Information:**

The online version contains supplementary material available at 10.1186/s40623-024-02116-2.

## Introduction

Carbonaceous chondrites contain the most primitive material in the solar system, and some have been aqueously altered to produce hydrous minerals, making them and their parent asteroids important volatile reservoirs that may have supplied water to the early Earth (King et al. 2019, 2022; Alexander et al. 2012). The ‘Mighei-like’ carbonaceous (CM) chondrites are a volatile-rich group of meteorites containing chondrules and/or their altered pseudomorphs, and have fine-grained matrices composed predominantly of serpentine with/without tochilinite (Fuchs et al. 1973; Bunch and Chan 1980; Rubin et al. 2007; Kimura et al. 2020). CM chondrite serpentine is composed of two varieties, Fe-rich and Mg-rich. These serpentines are usually not pure Fe-, Mg-endmembers and in studies of carbonaceous meteorites they are usually referred to as cronstedtite and lizardite, respectively (Howard et al. 2009). Many CM chondrites are polymict breccias and differ significantly in their degree of aqueous alteration (Lentfort et al. 2021; Rubin et al. 2007; Alexander et al. 2013; Howard et al. 2015; Kimura et al. 2020), with some meteorites showing this variance between clasts (Lentfort et al. 2021). Certain meteorites have also been thermally modified after aqueous alteration over short time periods (hours to years) in a process known as post-hydration heating (Nakamura 2005; Nakato et al. 2008). This process significantly affected the volatile contents of CM chondrites and their parent asteroids; it is thus important to understand how post-hydration heating progresses, including the temperatures of key mineral transitions (e.g., tochilinite decomposition, serpentine dehydroxylation) as this will yield more information on the role of C-complex asteroids as volatile sources.

Previous studies of post-hydration heating of CM chondrites have been able to determine much about the process and its products (e.g., Nakamura 2005; Lindgren et al. 2020; Nakato et al. 2008; Akai 1988, 1992; Velbel and Zolensky 2021). Tochilinite can decompose at temperatures below 300 °C and recrystallizes into a combination of troilite and magnetite; serpentine breaks down above 300 °C, eventually recrystallizing into olivine when temperatures exceed 500 °C; and oldhamite is a product of calcite decomposition at high temperatures (e.g., ≥ 400 °C) (Nakamura 2005; Lindgren et al. 2020; Nakato et al. 2008; Akai 1988, 1992; Velbel and Zolensky 2021; Haberle and Garvie 2017; Fuchs et al. 1973; Lee et al. 2016).

Despite the plethora of work done, there is still much unknown about post-hydration heating. Its cause and duration remain debated, and the temperatures of some phase transitions (e.g., tochilinite) are still poorly constrained. In addition, thermal decomposition of phases in polymineralic carbonaceous meteorites progresses differently than observed in the monomineralic terrestrial samples, thus limiting the applicability of terrestrial analogues (Velbel and Zolensky 2021). Given the variation in petrography and mineralogy that CM chondrites display (e.g., Howard et al. 2011, 2015), it is possible that their composition after aqueous processing but before thermal alteration (e.g., mineralogy, chemistry) may play a role in how post-hydration heating progresses.

There are many different components that could affect mineral transitions during post-hydration heating. Serpentine is a major constituent of most CM chondrites and differs significantly in abundance and composition depending on a given meteorite’s degree of aqueous alteration (Rubin et al. 2007; Howard et al. 2015). When serpentine decomposes, it releases water (Ball and Taylor 1963), which in turn may affect its atmospheric environment. Volatile-bearing phases that are stable at high temperatures (e.g., talc) may be more likely to form in a more hydrous atmosphere. The starting composition of serpentine could affect the anhydrous silicates that form from its recrystallization (e.g., Fe-rich serpentine producing Fe-rich anhydrous silicates). In addition, some CM chondrites contain more S-bearing phases (e.g., sulfides, tochilinite) than others (Howard et al. 2011, 2015) and when heated, should produce a greater amount of S-bearing secondary minerals (e.g., oldhamite, troilite).

To better understand post-hydration heating and its effects, we have experimentally heated two CM chondrites that differ in their degree of aqueous alteration and have no evidence for prior post-hydration heating: Allan Hills (ALH) 83100 (temperature range 200–950 °C) and Murchison (temperature range 300–750 °C). Both experiments used 25 °C temperature steps, and at each step we collected high-resolution in situ X-ray diffraction (XRD) data using synchrotron radiation. High-resolution XRD detectors, such as the multi-analyzing crystal detectors on the I11 beamline at Diamond Light Source, are particularly advantageous in studying mineral reactions in complex samples, like CM chondrites, as they allow for the detection of trace phases (e.g., ≤ 0.1 vol.%) and the separation of XRD peaks that are close together in 2ϴ, allowing phases with peak positions less than 0.05° apart to be distinguished (Thompson et al. 2009). We were able to study mineral transitions due to post-hydration heating at a fine scale and determine which mineral transitions differ depending on the meteorite’s initial composition.

## Samples

Samples of two CM chondrites, ALH 83100 (CM1/2) and Murchison (CM2), were experimentally heated. They differ in both degree of aqueous alteration and mineralogy as summarized in Table 1. Fe-rich and Mg-rich serpentines in these meteorites are not pure endmembers but are referred to as cronstedtite and lizardite for simplicity, both here and in the sources summarized in Table 1 (Rubin et al. 2007; Howard et al. 2011).

**Table 1:** Summary of the bulk properties of ALH 83100 and Murchison

	ALH 83100	Murchison
Petrologic subtype^a^	2.1	2.5
Lizardite (vol. %)^b^	62.4	22.2
Cronstedtite & tochilinite (vol.%)^b,c^	24.2	50.3
Olivine (vol. %)^b^	8.7	15.1
Orthopyroxene (vol. %)^b^	0.7	8.3
Sulfides (vol. %)^b^	1.0	1.8
Magnetite (vol. %)^b^	1.7	1.1
Calcite (vol. %)^b^	1.2	1.2
Types of carbonates present^d^	Calcite (Type 1b), dolomite	Calcite (Type 1a & 2a), aragonite

^a^Rubin et al. (2007)

^b^Howard et al. (2011)

^c^Howardet al. (2011) does not separate cronstedtite and tochilinite when determining their abundance

^d^Lee et al. (2014)

### ALH 83100

ALH 83100 is a highly aqueously altered CM chondrite (petrologic subtype 2.1; Rubin et al. 2007; Rubin 2015) whose modal mineralogy determined by Howard et al. (2011) is summarized in Table 1. Howard et al. (2011) was unable to quantify tochilinite; however, McSween (1987) estimates that the matrix contains ~ 8 wt.% tochilinite. A sample (ALH 83100,276) from the National Aeronautics and Space Administration (NASA) Antarctic Meteorite collection was acquired and powdered for XRD analysis. 100 mg was loaded and sealed into a 0.7 mm quartz capillary under argon gas to ensure that it was heated in an inert atmosphere.

### Murchison

Murchison is a moderately aqueously altered CM chondrite (petrologic subtype 2.5; Rubin et al. 2007; Rubin 2015). Its modal mineralogy determined by Howard et al. (2011) is summarized in Table 1. McSween (1987) estimates that the matrix of Murchison is composed of ~ 15 wt.% tochilinite. A sample of Murchison was acquired from Skyfall meteorites (Skyfall Meteorites 2024) and powdered for XRD analysis. 100 mg was loaded and sealed into a 0.7 quartz capillary under an inert argon atmosphere.

## Methods

Experimental heating and XRD data acquisition was conducted at the I11 beamline at Diamond Light Source. The I11 beamline is equipped with a U22 90 pole in vacuum undulator, a Si (111) double crystal monochromator, and a pair of harmonic rejection mirrors. Multi-analyzer crystal detectors were used to collect high resolution XRD patterns at a constant speed with Δ2*ϴ* = 0.001°. The detection limit of these multi-analyzer crystals is ≤ 0.1 vol.%. For this experiment, each XRD pattern took 1 h to collect, and the wavelengths used were 0.826890 Å with a zero error of -0.00719° for ALH 83100, and 0.823845 Å with a zero error of 0.00424° for Murchison. Wavelengths were calibrated against the National Institute of Standards and Technology Si reference powder SRM640c. For more information regarding beamline specifications, see Thompson et al. (2009).

Each sample in its capillary was placed on a capillary spinner at the center of the I11 diffractometer and spun for the duration of the experiment. A Cyberstar hot air blower was used to heat the samples at a rate of 12 °C per minute between set point temperatures, with the stability at each setpoint temperature ≥ 200 °C being ≤ 1 °C. An XRD pattern at room temperature was collected for each sample prior to heating. ALH 83100 was heated 200–950 °C, while Murchison was heated 300–750 °C. Both meteorites were heated at 25 °C temperature steps. Murchison was heated at a smaller temperature range due to its experiment being allotted less beamtime than the ALH 83100 experiment.

At its first temperature step of 200 °C, ALH 83100 was held for 60 min prior to data collection. After the XRD pattern for that temperature step had been collected, ALH 83100 was heated 25 °C to the next temperature step, wherein it was held again for another 60 min prior to data collection. This process was repeated for each temperature step until 950 °C was reached. After the 950 °C temperature step, ALH 83100 was cooled to room temperature, wherein another XRD pattern was collected. With both holding times and measurement times accounted for, ALH 83100 spent a total of 120 min at each temperature step.

Murchison’s heating regime differed slightly from ALH 83100. Due to a minor programming issue, Murchison was held for 90 min at its first temperature step of 300 °C prior to data collection. Murchison was then heated 25 °C to its next temperature step. To ensure that the full range of temperatures were covered in the allotted beamtime, it was held at subsequent temperature steps for 55 min prior to data collection. This difference in heating regime with ALH 83100 likely had a minimal effect on the mineral transitions observed. After the 750 °C temperature step, Murchison was brought down to room temperature and another XRD pattern was collected. With holding times and measurement times summed, Murchison spent 150 min at the 300 °C temperature step and 115 min at all other temperature steps.

All XRD patterns were processed with a combination of Rigaku Smartlab II (Rigaku 2024), Topas 4.2 (Coelho 2018), and Dawn 2.35.0 software (Basham et al. 2015; Filik et al. 2017). Mineral identification was conducted by matching peak positions to Crystallography Open Database (COD) mineral cards (Vaitkus et al. 2021, 2023; Merkys et al. 2016, 2023; Quirós et al. 2018; Gražulis et al. 2009, 2012, 2015; Downs and Hall-Wallace 2003).

## Results

### ALH 83100

The room temperature XRD pattern (Fig. S1) shows that ALH 83100 is composed mainly of lizardite (COD card 9001091) and cronstedtite (COD card 9005755), with minor amounts of forsterite (COD card 9000267), enstatite (COD card 9001597), calcite (COD card 9016706), tochilinite (COD card 9009525), magnetite (COD card 9000926), pyrrhotite (COD card 5000091), and pentlandite (COD card 1011181). This mineralogy is consistent with previous XRD studies of ALH 83100 (Howard et al. 2011; Lindgren et al. 2020). All XRD patterns for ALH 83100 can be viewed in Figs. S3–S10.

#### Sulfides

**Fig. 1:**
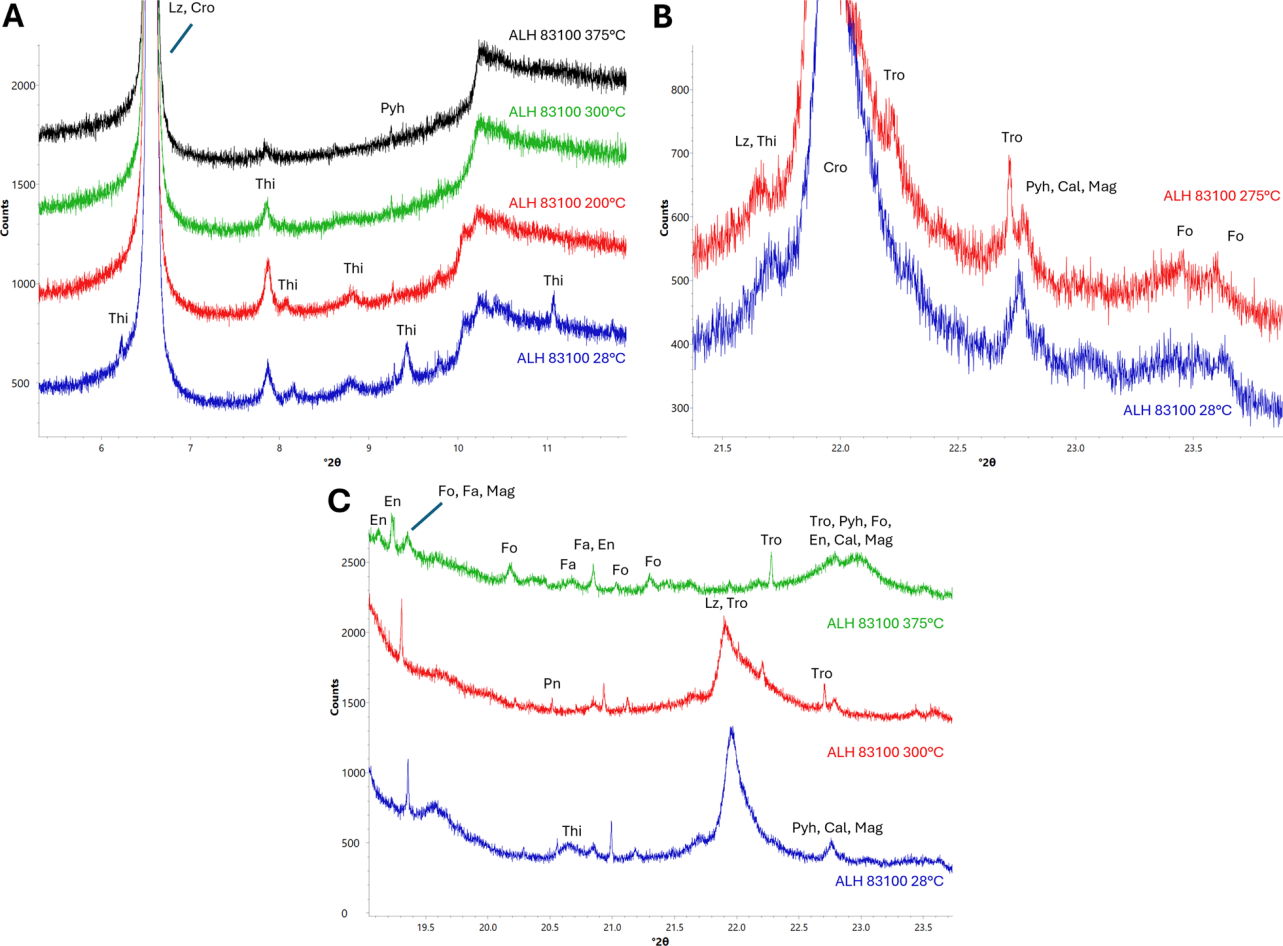
XRD patterns showing changes in ALH 83100 sulfides due to heating. Labelled minerals are tochilinite (Thi), lizardite (Lz), cronstedtite (Cro), pyrrhotite (Pyh), enstatite (En), pentlandite (Pn), forsterite (Fo), fayalite (Fa), magnetite (Mag), and troilite (Tro). **a** Thi decomposition. At 200 °C, many Thi peaks weaken or disappear. At 300 °C, nearly all Thi peaks are gone except for a single peak at *d* = 6.02 Å (13.3° 2*ϴ*). At 375 °C this last peak disappears. **b** Tro crystallization. At 275 °C, Tro peaks appear. **c** At 675 °C, Pn peaks weaken, with many disappearing

Between room temperature and 200 °C some, but not all, tochilinite peaks began to weaken and disappear. The tochilinite peaks that weakened were at *d* = 5.12 Å (9.3° 2*ϴ*), 5.03 Å (9.5° 2*ϴ*), and 4.28 Å (11.1° 2ϴ). Most tochilinite peaks, with the exception of those associated with intergrown cronstedtite at *d* = 6.02 Å (7.87° 2*ϴ*) (Nakamura and Nakamuta 1996), do not weaken until 275 °C. All tochilinite peaks disappear by 300 °C (Fig. 1a). The *d* = 6.02 Å (7.87° 2*ϴ*) tochilinite peak disappears by 375 °C. Troilite (COD card 9004036) peaks appear at 275 °C and strengthen for the rest of the experiment (Fig. 1b). Pentlandite peaks begin to weaken at 675 °C (Fig. 1c).

#### Mg- and Fe-silicates

**Fig. 2:**
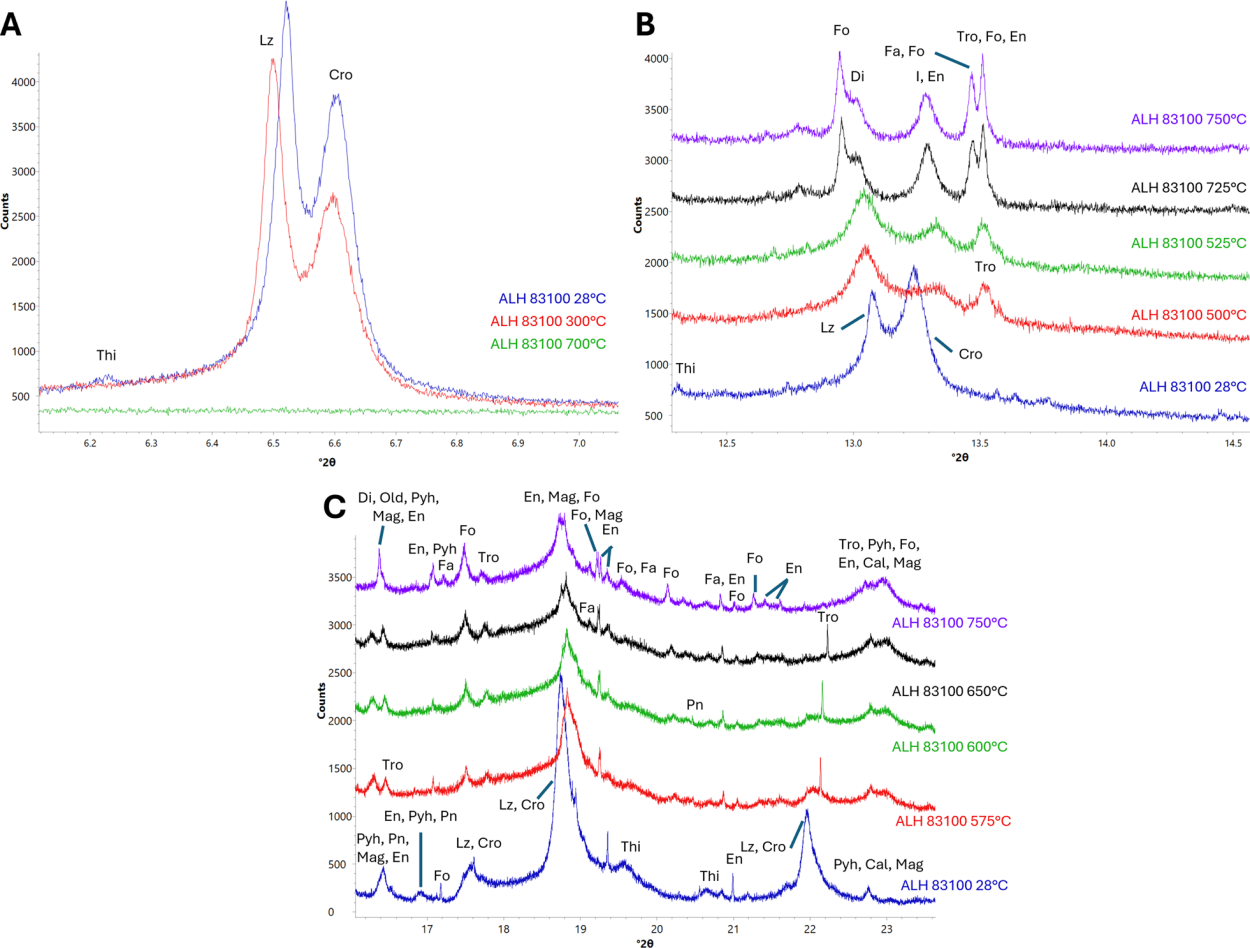
XRD patterns showing changes experienced by silicates in ALH 83100 due to heating. Labelled minerals are tochilinite (Thi), lizardite (Lz), cronstedtite (Cro), forsterite (Fo), diopside (Di), an unmatched phase (I), enstatite (En), fayalite (Fa), troilite (Tro), pentlandite (Pn), pyrrhotite (Pyh), calcite (Cal), and magnetite (Mag). **a** Serpentine decomposition. Lz and Cro peaks begin weakening at 300 °C and disappear by 700 °C. Shifting of Lz and Cro peaks between 28 °C and 300 °C is due the thermal expansion of their crystal lattices. **b** Changes experienced by unmatched phase and Di. A peak at *d* = 3.56 Å (13.3° 2*ϴ*) begins strengthening at 525 °C until 750 °C is reached, wherein it begins weaking. Di is also shown appearing at 725 °C. **c** Crystallization of secondary En, Fo, and Fa. Some but not all En peaks begin strengthening at 575 °C. All En peaks strengthen later at 750 °C. Fo peaks strengthen at 600 °C and Fa peaks appear at 650 °C

Weakening of serpentine peaks begins at 300 °C, with cronstedtite peaks showing greater amounts of change in intensity, indicating a faster decomposition rate. As heating progresses, cronstedtite and lizardite peaks become more difficult to differentiate from one another due to peak broadening. Small serpentine peaks were still present at 650 °C, and all serpentine peaks disappear by 700 °C (Fig. 2a).

At 525 °C, a peak at *d* = 3.56 Å (13.3° 2*ϴ*) appears and strengthens until 750 °C, wherein it begins to weaken (Fig. 2b). It is not matched to any minerals and is likely associated with one of the two transitional structures (dehydroxylate I and II) between serpentine and secondary olivine and enstatite that develop during serpentine dehydroxylation. These transitional structures are discussed further in the serpentine decomposition section of the discussion.

Some enstatite peaks begin to strengthen at 575 °C, while forsteritic olivine peaks noticeably strengthen at 600 °C. Applying the Schwab and Küstner method of determining Fo content described by Di Cecco et al. (2022) to the room temperature pattern taken after heating to account for any shifting of peaks due to heating, the d-spacing of the (1 3 0) peak is 2.77 Å, indicating that this olivine is ~ 93% forsterite (Di Cecco et al. 2022). Fayalitic olivine (COD card 9000469) peaks appear at 650 °C (Fig. 2c). This olivine has a d-spacing of 2.80 Å at room temperature, indicating a forsterite content of ~ 47%. Enstatite peaks that showed no change at 575 °C begin strengthening at 750 °C (Fig. 2c). Both enstatite and olivine peaks strengthen for the rest of the experiment. No olivine peaks associated with intermediate compositions were observed.

#### Ca-bearing minerals

**Fig. 3:**
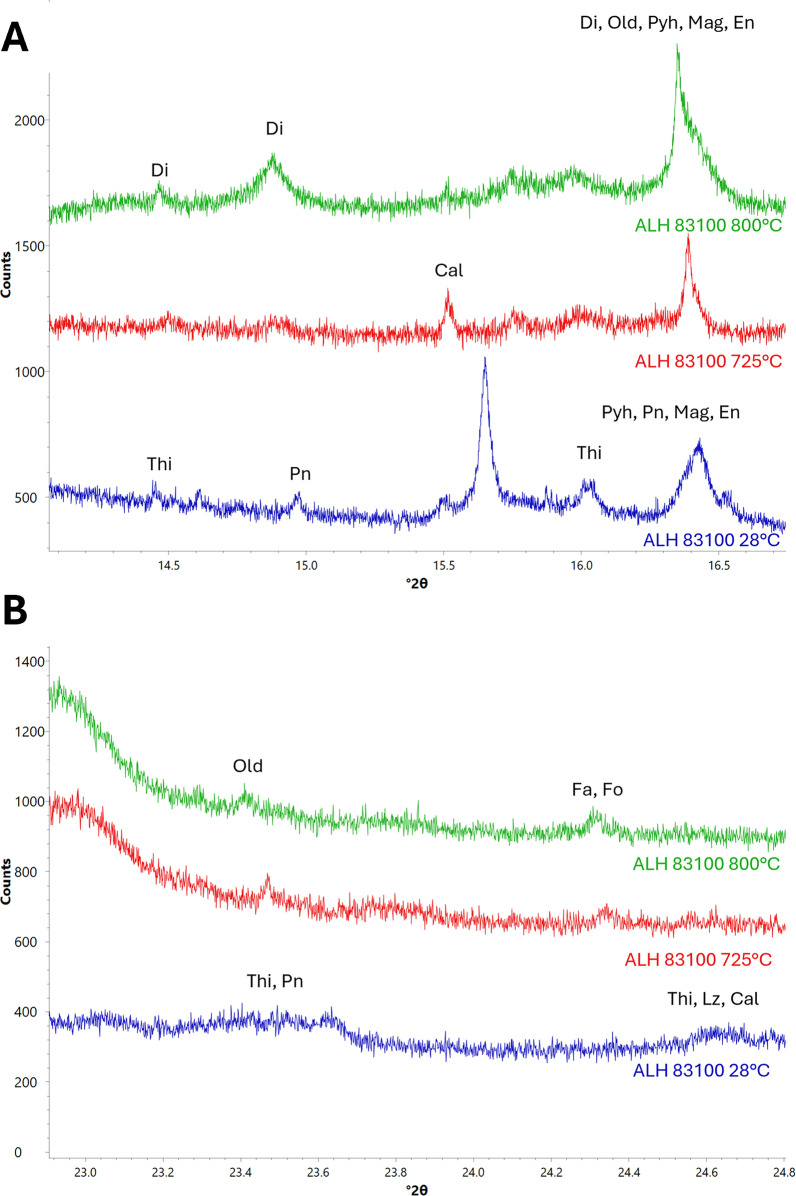
XRD patterns showing changes experienced by Ca-bearing minerals in ALH 83100 due to heating. Labelled minerals are diopside (Di), oldhamite (Old), pyrrhotite (Pyh), calcite (Cal), tochilinite (Thi), pentlandite (Pn), fayalite (Fa), forsterite (Fo), enstatite, (En), magnetite (Mag), and lizardite (Lz). **a** Cal decomposition. Cal peaks begin weakening at 725 °C and disappear by 800 °C. **b** Old crystallization. Old appears at 725 °C

Calcite peaks begin weakening at 725 °C and disappear by 800 °C. At 725 °C, peaks associated with oldhamite (COD card 9008606) and diopside (COD card 1000008) appear (Fig. 3) and strengthen for the rest of the experiment.

### Murchison

The room temperature XRD pattern for Murchison (Fig. S2) showed significant amounts of lizardite (COD card 9000848), cronstedtite (COD card 9000019), forsterite (COD card 9000322), and enstatite (COD card 1000047), as well as minor amounts of tochilinite (COD card 9009525), magnetite (COD card 1011084), pyrrhotite (COD card 9000279), pentlandite (COD card 1011181), and calcite (COD card 4502443). This mineralogy is consistent with published reports (e.g., Howard et al. 2011). All XRD patterns for Murchison can be viewed in Figs. S11–S15.

#### Sulfides and oxides

**Fig. 4:**
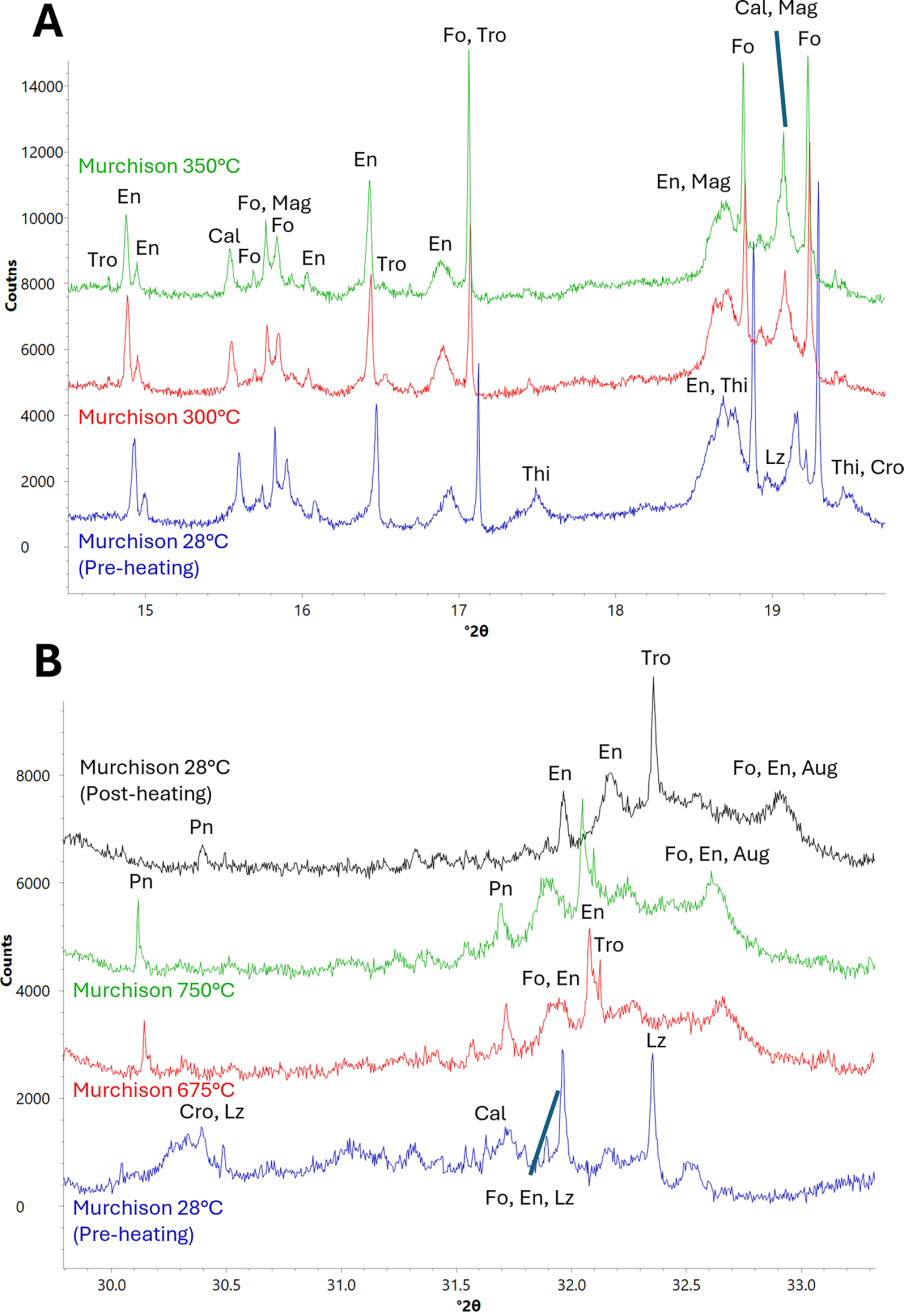
XRD patterns showing changes experienced by sulfides in Murchison. Minerals labelled are troilite (Tro), enstatite (En), calcite (Cal), forsterite (Fo), magnetite (Mag), tochilinite (Thi), lizardite (Lz), cronstedtite (Cro), pentlandite (Pn), and augite (Aug). **a** Decomposition of Thi and crystallization of secondary Tro and Mag. Thi peaks disappear at 300 °C, while at the same time Tro peaks appear. Mag peaks (e.g., 15.8°, 18.7°, and 19.1° 2*ϴ*) start strengthening at 350 °C. **b** Pn decomposition. Pn peaks start weakening at 675 °C, with peak weakening accelerating during the cooldown to room temperature. Tro peaks also start strengthening significantly with Pn decomposition

All tochilinite peaks disappear at the first heating step of 300 °C, while at the same temperature troilite (COD card 9007651) peaks appear. Magnetite peaks strengthen at 350 °C (Fig. 4a).

Pentlandite peaks begin weakening at 675 °C. After 750 °C, when Murchison has been cooled to room temperature, troilite showed a dramatic increase in peak intensity (Fig. 4b).

#### Mg- and Fe-silicates

**Fig. 5:**
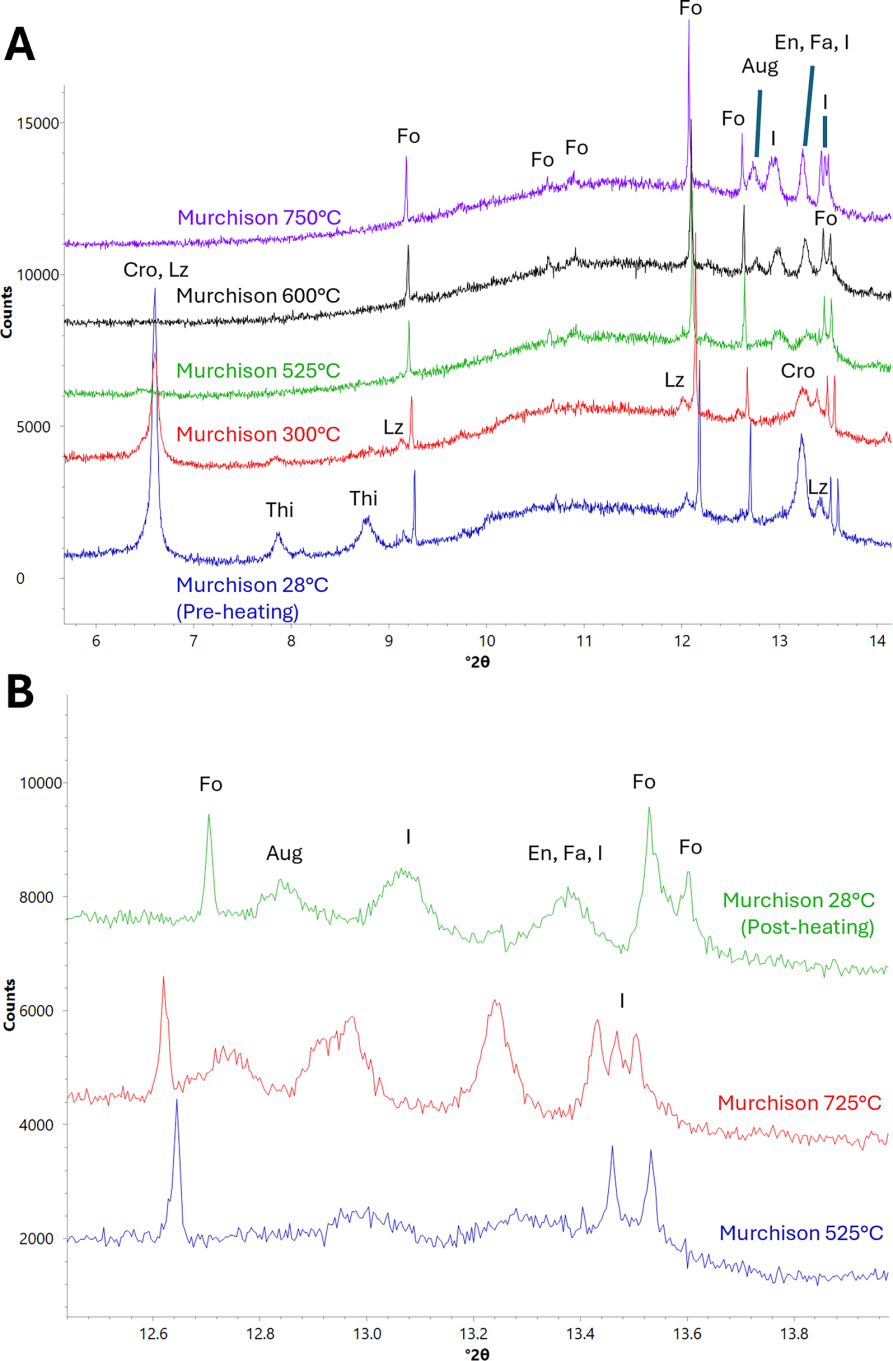
XRD patterns showing changes as a result of serpentine decomposition in Murchison. Minerals labelled are cronstedtite (Cro), lizardite (Lz), tochilinite (Thi), forsterite (Fo), augite (Aug), enstatite (En), fayalite (Fa), and an unmatched phase (I). **a** Serpentine decomposition and crystallization of secondary silicates. Both Cro and Lz (e.g., see peaks at 9.1°, 12.0°, 12.2°, 13.2°, and 13.3° 2*ϴ*) begin decomposition at 300 °C, with peaks associated with Cro (e.g., 13.2° 2*ϴ*) weakening at a faster rate. All serpentine peaks disappear at 525 °C. At 525 °C, peaks associated with an unmatched phase appear and strengthen. At 600 °C, Fo peaks strengthen, and Fa peaks appear. En peaks strengthen at 750 °C. **b** Weakening of the peaks associated with the unmatched phase, which becomes most apparent during cool down to room temperature

Serpentine peaks begin weakening at the first heating step of 300 °C. Cronstedtite peaks show a greater degree of peak weakening compared to lizardite peaks, indicating faster decomposition. As heating progresses, cronstedtite and lizardite peaks become more difficult to differentiate from one another due to peak broadening. All serpentine peaks disappear by 525 °C (Fig. 5a).

At 525 °C, three unmatched peaks appear at d-spacings of 3.53 Å (13.4° 2*ϴ*), 3.56 Å (13.2° 2*ϴ*), and 3.67 Å (12.7° 2*ϴ*) (Fig. 5a) and strengthen until Murchison is brought down to room temperature, wherein the 3.53 Å and 3.67 Å peaks weaken and the 3.56 Å peak disappears (Fig. 5b).

Forsteritic olivine peaks start strengthening at 600 °C and continue to do so for the rest of the experiment (Fig. 5a). This olivine has a final d-spacing of 2.77 Å when cooled to room temperature, indicating a forsterite content of ~ 93% (Di Cecco et al. 2022). A fraction of enstatite peaks start strengthening at 600 °C, while all enstatite peaks begin strengthening at the final temperature step of 750 °C (Fig. 5a).

#### Ca-bearing minerals

**Fig. 6:**
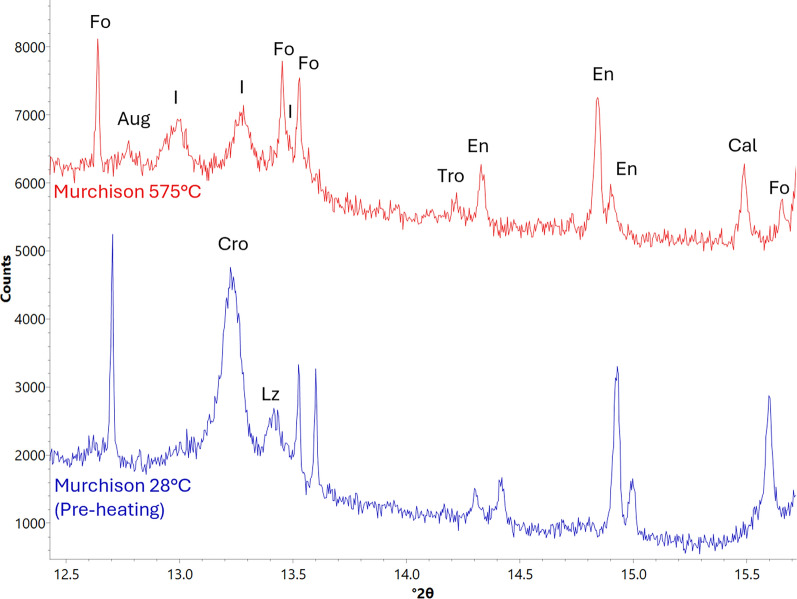
XRD patterns showing changes experienced by Ca-bearing phases in Murchison during heating. Labelled minerals are forsterite (Fo), augite (Aug), an unmatched phase (I), troilite (Tro), enstatite (En), calcite (Cal), cronstedtite (Cro), and lizardite (Lz). At 575 °C, Cal peaks start weakening and peaks associated with Aug appear

Calcite peaks begin weakening at 575 °C and continue to do so for the rest of the experiment although they never disappear. Augite (COD card 1200006) peaks also appear at 575 °C (Fig. 6) and continue strengthening for the rest of the experiment. No oldhamite was observed.

## Discussion

**Fig. 7:**
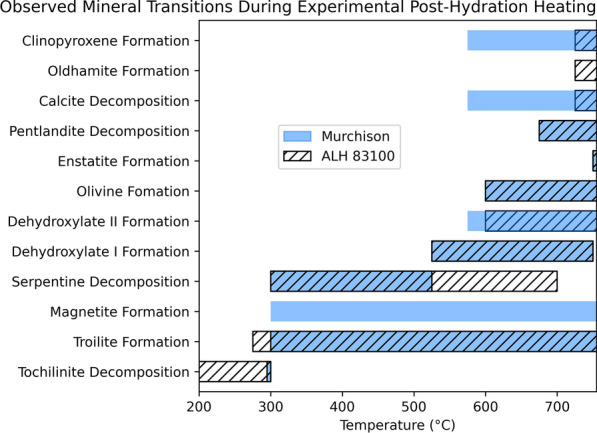
Mineral transitions observed in ALH 83100 and Murchison during experimental post-hydration heating

Both ALH 83100 and Murchison experienced a variety of mineralogical changes (summarized in Table 2 and Fig. 7) during experimental heating. Many of these changes (e.g., tochilinite decomposition temperature) were consistent between both meteorites, but some differed significantly (e.g., calcite decomposition). We discuss both how specific phase transitions progress as well as what may have caused the differences between the two meteorites.

**Table 2:** Temperatures at which post-hydration heating mineral transitions occur in this study compared with literature reports

	ALH 83100	Murchison	Literature	References
Tochilinite decomposition	200–300 °C	≤ 300 °C	120–300 °C	a, b, c
Troilite formation	≥ 275 °C	≥ 300 °C	≥ 245 °C	a
Magnetite formation	n.d	≥ 350 °C	≥ 250 °C	c
Serpentine decomposition	300–700 °C	300–525 °C	300–500 °C	d, e
Dehydroxylate I	525–750 °C	525–750 °C	400–600 °C	d, f, g
Dehydroxylate II	≥ 600 °C	≥ 575 °C	600–800 °C	g
Olivine formation	≥ 600 °C	≥ 600 °C	≥ 500 °C	d, e
Pentlandite decomposition	≥ 675 °C	≥ 675 °C	≥ 610 °C	h, i
Enstatite formation	≥ 750 °C	≥ 750 °C	≥ 800 °C	d
Calcite decomposition	725–800 °C	≥ 575 °C	440–800 °C	h, j, k, l
Oldhamite formation	≥ 725 °C	n.d	≥ 400 °C	m, n
Clinopyroxene formation	≥ 725 °C	≥ 575 °C	≥ 800 °C	m, n

^a^Fuchs et al. (1973)

^b^Zolensky et al. (1997)

^c^Tonui et al. (2014)

^d^Akai (1992)

^e^Ball and Taylor (1963)

^f^Akai(1988)

^g^Mackenzie and Meinhold (1994)

^h^Nakato al. (2008)

^i^Kullerud (1963)

^j^Karunadasa et al. (2019)

^k^Lee et al. (2019)

^l^Rodriguez-Navarro et al. (2009)

^m^Wang and Thomson (1995)

^n^Lindgren et al. (2020)

### Tochilinite decomposition

Tochilinite [Fe^2+^_5–6_,(Mg,Fe^2+^)_5_S_6_(OH)_10_] is a hydrous sulfide composed of intergrown layers of mackinawite, brucite, and/or amakinite (Palmer and Lauretta 2011), and its decomposition temperature is poorly constrained. The lowest temperature tochilinite has been observed decomposing at was 245 °C in experiments conducted by Fuchs et al. (1973), where they held samples of Murchison at that temperature for 7.7 days.

Here, we observed tochilinite’s complete decomposition occurring by 300 °C in both ALH 83100 and Murchison, with all peaks apart from the one related to intergrowths with cronstedtite disappearing (Figs. 1a and 4a). This is at a higher temperature than was observed by Fuchs et al. (1973); however, we were heating ALH 83100 and Murchison at much shorter timescales. In addition, in ALH 83100, secondary phases that result from tochilinite decomposition (e.g., troilite) crystallized at 275 °C (Fig. 1b), showing that decomposition had begun at ≤ 275 °C. It is likely if we had held ALH 83100 at each step for longer (e.g., days to weeks), tochilinite would have completely decomposed below 300 °C.

For ALH 83100 specifically, some tochilinite peaks weakened at 200 °C; however, a portion of its peaks showed no change, remaining at a constant intensity until complete decomposition at 300 °C (Fig. 1a). Thus, tochilinite appears to be undergoing partial decomposition at 200 °C. As tochilinite has intergrown layers, it is likely that some, but not all, of these layers were decomposing at 200 °C. Brucite can decompose at ~ 200 °C (Wang et al. 1998), while mackinawite requires heating to at least 210 °C to experience any changes, wherein it transforms into pyrrhotite, with the temperature at which this occurs varying with its composition (Sarkar 1971). It is possible that mackinawite may transform into troilite under inert and reducing conditions.

We propose that tochilinite’s decomposition due to post-hydration heating is a two-stage process. The first stage is the breakdown of its brucite and/or amakinite layers, which may occur at temperatures as low as 200 °C. The second stage would be the transformation of its mackinawite layers, which requires a minimum temperature of 210 °C, though higher temperatures may be needed depending on its composition. This two-stage process would only require tochilinite to become partially amorphous, which is consistent with the observations of this study, wherein secondary phases (e.g., troilite) appeared prior to complete tochilinite decomposition (Fig. 1).

Both meteorites show the same temperature for complete tochilinite decomposition regardless of their starting composition. As a result of tochilinite decomposition, both ALH 83100 and Murchison show secondary troilite at 275 °C and 300 °C, respectively (Figs. 1b and 4a). The two meteorites share similar crystallization temperatures for troilite, regardless of starting composition. Secondary magnetite was detected in Murchison at 350 °C. We did not detect secondary magnetite in ALH 83100, but it was observed by Lindgren et al. (2020), who heated chips of ALH 83100 to 400 °C. However, Lindgren et al.’s (2020) detection was through Raman spectroscopy with the XRD determined vol.% of magnetite being similar between unheated and heated samples, especially when sample heterogeneity is considered. It is, therefore, likely that secondary magnetite did occur within heated ALH 83100, but in quantities below the XRD detection limit of 0.1 vol.%.

### Pentlandite decomposition

Pentlandite is reported to have a decomposition temperature of 610 °C (Kullerud 1963; Nakato et al. 2008; Etschmann et al. 2004), with this estimate being based on the decomposition of terrestrial pentlandite (Kullerud 1963). We observed pentlandite decomposition at 675 °C in both ALH 83100 and Murchison (Figs. 1c and 4b); however, the higher temperature of decomposition observed here is likely the result of the short timescales used in this study. Both ALH 83100 and Murchison show similar decomposition rates for pentlandite, regardless of their starting composition.

### Serpentine decomposition

Lizardite and cronstedtite peaks start weakening at 300 °C in both ALH 83100 and Murchison, with cronstedtite peaks weakening at a faster rate than those of lizardite (Figs. 2a and 5a). This decomposition temperature and rate is consistent with previous reports for serpentine decomposition in CM chondrites (Akai 1992). Serpentine decomposition begins at the same temperature (~ 300 °C) regardless of the meteorite’s starting composition.

Serpentine completely decomposed at different temperatures in ALH 83100 and Murchison, happening at 700 °C and 525 °C, respectively (Figs. 2a and 5a). Thus, the starting composition and texture has an effect on serpentine decomposition rate. This could be due to grain size as experimental studies on terrestrial serpentine have shown that smaller grain sizes lead to faster decomposition rates (Alizadehhesari et al. 2012). Murchison serpentine may be finer grained than ALH 83100 due to it experiencing aqueous alteration for a shorter duration and/or at a lower temperature, resulting in less crystal growth. The latter is consistent with clumped isotope data in Clog et al. (2024), which shows that carbonates in Murchison formed at lower temperatures than in ALH 83100 (5–22 °C vs. 51–101 °C, respectively).

Chemical composition may have also played a role in the differing decomposition rates between ALH 83100 and Murchison. Here, we observed cronstedtite showing a greater degree of peak weakening compared to lizardite, indicating a faster decomposition rate. The decomposition rate of cronstedtite is dependent on redox conditions, with its breakdown occurring more quickly under reducing than oxidizing conditions (Mackenzie and Berezowski 1981). Other studies on serpentine decomposition using faster heating rates (e.g., for differential thermal analysis) have shown cronstedtite dehydroxylating at a lower temperature than lizardite (Caillère and Hénin 1957). Cronstedtite, therefore, decomposes at a faster rate than lizardite. Murchison has a greater proportion of cronstedtite to lizardite than ALH 83100 (Table 1; Howard et al. 2011), which led to the breakdown of all serpentine happening sooner during heating. CM chondrites that are less aqueously altered and have greater proportions of cronstedtite require shorter heating durations for the complete breakdown of their serpentine matrices.

At ~ 525 °C in both the ALH 83100 and Murchison experiments, peaks appeared at d-spacings of 3.53 Å, 3.56 Å, and 3.67 Å (Figs. 2b and 5a). Although some of these peaks overlapped with other anhydrous silicates (e.g., enstatite), another crystalline material was determined to be present as these peaks weakened at ~ 750 °C for both meteorites (Figs. 2a and 5b). No mineral cards for phases that would form at 525 °C but decompose at 750 °C matched. This phase likely belongs to one of two transitional structures, known as dehydroxylate I and II, that have been previously reported to form as intermediary phases between serpentine and secondary anhydrous silicates during serpentine decomposition (Dlugogorski and Balucan 2014; Mackenzie and Meinhold 1994). Furthermore, transitional structures from serpentine decomposition have been reported to occur at 400–600 °C in heated CM chondrites (Akai 1988, 1992). Dehydroxylate I and II crystallize into olivine and enstatite, respectively (Dlugogorski and Balucan 2014; Mackenzie and Meinhold 1994). The transitional structure observed here is likely to be dehydroxylate I, which forms prior to dehydroxylate II during serpentine decomposition (Dlugogorski and Balucan 2014; Mackenzie and Meinhold 1994). Dehydroxylate I’s consistent appearance and disappearance in both meteorites shows that its occurrence is not dependent on their original composition.

During decomposition, serpentine breaks down into Mg/Fe-rich and Si-rich regions, with olivine crystallizing from the Mg/Fe-rich regions (Ball and Taylor 1963; Dlugogorski and Balucan 2014; Mackenzie and Meinhold 1994). These Mg/Fe-rich regions have similarities to the original serpentine octahedral layer from which they are derived and are not subjected to the same degree of reorganization as the Si-rich regions, leading them to be relatively “immobile” (Dlugogorski and Balucan 2014). As a result of the immobility of the Mg/Fe-rich regions, it is likely that meteorites with distinct populations of cronstedtite and lizardite, like many CM chondrites including ALH 83100 and Murchison (Rubin et al. 2007), should produce two distinct populations of olivine, with cronstedtite forming fayalitic olivine and lizardite producing forsteritic olivine.

Crystallization temperatures for secondary forsteritic olivine are consistent between ALH 83100 and Murchison, with the strengthening of its peaks starting at 600 °C (Figs. 2c and 5a). This observation is also congruent with the 500–750 °C crystallization temperature reported for olivine from serpentine decomposition (Ball and Taylor 1963; Akai 1992). Initial meteorite composition has no effect on the formation temperature of secondary forsterite.

Fayalitic olivine formation due to serpentine decomposition differs between both meteorites. In ALH 83100 it occurs after forsteritic olivine formation at 650 °C in small quantities, while in Murchison it does not appear at all.

Experimental studies investigating the thermal decomposition of cronstedtite have shown that it forms fayalitic olivine at the same temperature as forsterite crystallization from the breakdown of lizardite (Mackenzie and Berezowski 1981; Akai 1992). Here, we observe fayalitic olivine forming at 650 °C in ALH 83100. In ALH 83100, fayalitic olivine was likely forming at the same time as forsteritic but had a slow formation rate and did not occur in quantities sufficient to be detectable by XRD until 650 °C.

Initial meteorite composition has an effect on whether or not fayalitic olivine appears in detectable quantities. In ALH 83100, although the most intense fayalitic olivine peak appeared at 650 °C, less intense peaks required more time to be apparent, with the (1 3 0) peak used to calculate its Fo content not appearing at a detectable intensity until 750 °C (Fig. 2c). The formation of fayalite is, therefore, likely a slower process than forsterite. Although XRD shows that Murchison has a greater amount of combined cronstedtite and tochilinite than ALH 83100 (Table 1; Howard et al. 2011), its matrix has a chemical composition consistent with having less total Fe-rich serpentine than ALH 83100 (McSween 1987). Despite having a higher cronstedtite-to-lizardite ratio, Murchison likely did not have a sufficient amount of cronstedtite to form fayalitic olivine in detectable quantities prior to the experiment’s completion. Given the slow formation rate of fayalite, it is possible that fayalitic olivine may have formed if Murchison had been heated for longer durations.

The crystallization of secondary enstatite proceeded similarly between ALH 83100 and Murchison. Some, but not all enstatite peaks started strengthening at 575 °C and 600 °C in ALH 83100 and Murchison, respectively, followed by the strengthening of all enstatite peaks at 750 °C in both meteorites (Figs. 2c, 5a, c). This result is congruent with other studies, wherein it was found that secondary enstatite does not always form from serpentine decomposition and when it does, it is in small quantities at higher temperatures, with olivine being the primary product (Ball and Taylor 1963; Akai 1992; Mackenzie and Meinhold 1994; Brindley and Hayami 1965). Experimental studies on terrestrial serpentines have shown that olivine crystallizes from the Mg/Fe-rich octahedral layer of serpentine (Dlugogorski and Balucan 2014), while the crystallization of enstatite requires the reorganization of silica tetrahedra derived from serpentine’s tetrahedral layer and the decomposition of the transitional structure, dehydroxylate II (Mackenzie and Meinhold 1994). The decomposition of serpentine favors the crystallization of olivine, with enstatite crystallization occurring at a slower rate (Brindley and Hayami 1965), observed at ≥ 800 °C in the majority of studies (Akai 1992; Nozaki et al. 2006; Mackenzie and Meinhold 1994; Dlugogorski and Balucan 2014). Given that not all enstatite peaks strengthened at 575–600 °C in both meteorites, the peak strengthening observed at those temperatures is likely not the result of enstatite crystallization but rather the formation of the transitional structure dehydroxylate II, which forms at a higher temperature than dehydroxylate I (Mackenzie and Meinhold 1994).

Full enstatite recrystallization began at 750 °C in both meteorites, indicating that the meteorite’s starting mineralogy had no effect on its temperature of formation. What is noteworthy here is that secondary enstatite was observed forming below the 800 °C temperature commonly reported in the literature (e.g., Akai 1992; Nozaki et al. 2006; Mackenzie and Meinhold 1994; Dlugogorski and Balucan 2014). The 750 °C secondary enstatite formation temperature observed here is likely the result of the fine 25 °C temperature steps and the high-resolution XRD used, allowing phases to be observed in low abundance with more precise constraints on their formation temperatures.

Our results show that the starting composition of a CM chondrite does not affect the temperatures at which secondary silicates crystallize from serpentine decomposition but does impact the relative quantities of minerals present, especially in regard to fayalitic olivine. Secondary olivine formed at 600 °C in this study, yet ALH 83100 and Murchison were heated for a timescale of hours. Other studies that have heated meteorites for longer timescales (e.g., days) observed the crystallization of secondary olivine at the lower temperature of 500 °C (e.g., Akai 1992; Ball and Taylor 1963). In contrast, Nozaki et al. (2006) who experimentally heated Murchison at a shorter timescale of 120 s observed secondary olivine occurring at 700 °C. The temperature at which secondary anhydrous silicates are observed forming at is dependent on heating duration.

The differences between this study and those utilizing differing timescales must be emphasized, especially when applying the knowledge gained from both this study and other experimental investigations to the analysis of naturally heated CM chondrites. A heating effect first observed at a high temperature in a sample heated for hours may also occur at lower temperatures if heating occurred for longer durations (e.g., days).

### Calcite decomposition

Literature estimates of calcite’s decomposition temperature vary. Some state that it decomposes at 700–800 °C (Karunadasa et al. 2019), while others posit that it breaks down at ~ 600 °C (Lee et al. 2019; Rodriguez-Navarro et al. 2009), and some estimates are as low as ~ 440 °C (Wang and Thomson 1995). Likewise, the results of this study are also variable, with calcite decomposing at 725 °C and 575 °C in ALH 83100 and Murchison, respectively (Figs. 2c, 3a, and 6).

The variability in calcite’s decomposition temperature is due to numerous factors, including, but not limited to, crystal size, morphology, impurities, and defects (Galan et al. 2013). Increased lattice strain has been directly related to lower calcite decomposition temperatures (Thompson et al. 2014). ALH 83100 and Murchison both contain calcite, but the populations are different (Table 3). ALH 83100 is more aqueously altered and contains type 1b calcites: equant crystals rimmed by sulfides (Lee et al. 2014). In addition, ALH 83100’s carbonates, as with many other highly altered CM chondrites, tend to be more Mg-rich, containing dolomite and Mg-rich calcite in addition to pure Ca-calcite (Zolensky et al. 1997; Rubin et al. 2007). ALH 83100 shows no sign of shock (Zolensky et al. 1997), and therefore, its calcite may be unstrained. ALH 83100 is also an Antarctic find (weathering grade: Be; Grossman et al. 1994) and could contain terrestrial calcite, which is known to occur for Antarctic CM chondrites (e.g., Tyra et al. 2007). In contrast, the less aqueously altered Murchison has type 1a and type 2 calcites; type 1a calcites are equant crystals rimmed by phyllosilicates, while type 2 calcites are polymineralic assemblages intergrown with sulfides (Lee et al. 2014). All of Murchison’s carbonates are pure Ca-carbonates (Rubin et al. 2007). In addition, Murchison has a shock stage of S1–S2 and shows strain in its silicates (Scott et al. 1992); it is, therefore, likely that its calcite is also strained. Although Murchison has experienced some weathering due its terrestrial age, it is a fall (Rubin et al. 2007), and is less likely to have terrestrial calcite. The calcites in ALH 83100 and Murchison differ in numerous aspects (Table 3), which have affected their decomposition temperature. Calcites from more altered CM chondrites like ALH 83100 may require higher temperatures to decompose; however, it is also possible that their differing impact histories (and thus different degrees of calcite lattice strain) are responsible. More experimental heating studies on other meteorites are required to confirm which factor(s) are responsible.

**Table 3:** Summary of differences between ALH 83100 and Murchison pertaining to calcite

	ALH 83100	Murchison	References
Degree of aqueous alteration	2.1	2.5	a, b
Calcite types	1b	1a, 2	c
Calcite associations	Sulfides	Phyllosilicates, sulfides	c
Mg-bearing calcites	Present	Absent	a, d
Other carbonates present	Dolomite	Aragonite	c
Shock-level	S1	S1–2	d, e
Year of fall	Unknown; Antarctic find	1969	f, g
Weathering grade	Be	W1–2	a, f

^a^Rubin et al. (2007)

^b^Rubin et al. (2015)

^c^Lee et al. (2014)

^d^Zolensky et al. (1997)

^e^Scott et al. (1992)

^f^Grossman et al. (1994)

^g^Moore (1970)

Regarding the products of calcite decomposition, ALH 83100 and Murchison differ. ALH 83100’s calcite produced both oldhamite and clinopyroxene, while Murchison’s calcite only produced clinopyroxene (Figs. 2c, 3b, and 6). As Murchison has a higher abundance of S-bearing phases (e.g., tochilinite) than ALH 83100 (Table 1; Howard et al. 2011; McSween 1987), and oldhamite is a sulfide, this difference in calcite decomposition products is likely not a direct result of bulk meteorite composition, but rather related to the oldhamite’s precursors (e.g., calcite). The contrasts between the two meteorites could be related to calcite’s decomposition temperature; however, Lindgren et al. (2020) observed oldhamite forming at 400 °C in ALH 83100. Another possibility is that whether or not oldhamite forms could be related to calcite type, with only calcites associated with sulfides producing oldhamite. Yet, the heated CM-like chondrite Sutter’s Mill contains a plethora of oldhamite grains, some of which are rimmed by olivine whose characteristics are consistent with it being produced from serpentine decomposition and whose precursors were probably type 1a calcite (Haberle and Garvie 2017), like that in Murchison. Calcite’s morphology is also unlikely to control its decomposition products. However, oldhamite requires more than just calcite to form; it also needs a sulfide. Both ALH 83100 and Murchison were heated in steps, with all of their tochilinite completely decomposing by 300 °C; much of tochilinite’s sulfur was likely consumed by the formation of secondary troilite. Both meteorites contain pentlandite which also decomposed here, albeit at a higher temperature (675 °C). We propose that ALH 83100’s calcite decomposed after pentlandite and reacted with its sulfur to form oldhamite, whereas Murchison’s calcite decomposed before pentlandite and thus the liberated calcium had no sulfur to react with. Clinopyroxene is, therefore, a ubiquitous product of calcite decomposition, while oldhamite likely only forms when calcite decomposes at higher temperatures and reacts with pentlandite (e.g., ≥ 610 °C) or potentially only in meteorites that have been rapidly heated, allowing calcite to react with other sulfides.

### Application to naturally heated meteorites

The results of this study are most comparable to meteorites that have experienced heating on the order of hours on their parent bodies. The fine temperature steps used have provided a detailed geothermometer for specific mineral transitions for heating timescales on the order of hours. However, timescale has a large impact on the heating effects observed in meteorites. Both heating duration and rate must be accounted for when comparing the results of this study to naturally heated meteorites to understand the geologic histories of their parent asteroids.

Here, serpentine started decomposing at 300 °C, but did not reach completion until 525–700 °C, which, in the case of ALH 83100, was after the formation of its products, like secondary olivine (Table 2). The coexistence of crystalline serpentine with secondary olivine in a naturally heated meteorite would indicate that it was heated to temperatures above 600 °C for a duration of only hours. Most moderately to highly heated CM chondrites described in the literature display either crystalline serpentine or secondary olivine and not both (e.g., Nakamura 2005; King et al. 2021). Other groups of meteorites that have experienced post-hydration heating, like the Yamato-type carbonaceous chondrites also do not show this coexistence of crystalline serpentine and secondary olivine (King et al. 2019). It is, therefore, apparent that natural post-hydration heating displayed by most meteorites occurred for durations longer than hours, and is likely on the order of days.

In this study, the transitional structures (e.g., dehydroxylate I, II) appeared at 525 °C, prior to any secondary silicates (Table 1), while other studies using longer durations found that the two coexisted (Akai 1988; Ball and Taylor 1963). A naturally heated meteorite with serpentine coexisting with transitional structures (e.g., dehydroxylate I and/or II) but not secondary silicates would not only again indicate short heating durations on the order of hours, but also indicate that it had experienced a narrow range of peak temperatures of 525–600 °C. An example of such a meteorite is the ungrouped carbonaceous Wisconsin Range 91600, which has both serpentine and one or both of its transitional structures but no secondary olivine (Tonui et al. 2014). Contrasting this, a meteorite with transitional structures (e.g., dehydroxylate I and/or II) and no serpentine, would indicate that it had experienced 500–600 °C of heating for durations longer than a few hours. An example of a CM chondrite that fits this description is Elephant Moraine 96029 (Lee et al. 2016).

Fayalitic olivine was found to form at a slower rate than forsteritic olivine, with this difference being most apparent at lower temperatures (e.g., ≤ 750 °C). Heated meteorites that have experienced temperatures below 750 °C (e.g., have no secondary enstatite), but have an abundance of secondary fayalitic olivine would, therefore, have experienced heating for more than a few hours. An example of a meteorite that have secondary fayalitic olivine, but no secondary enstatite is CM chondrite Yamato (Y)-82054 (King et al. 2021), which has, therefore, likely experienced heating for more than a few hours. In contrast, a heated meteorite that has secondary olivine that is mainly forsteritic in composition, likely experienced heating at temperatures ≥ 575 °C for short durations, similar to those used in this study (e.g., hours). Due to primary olivine in CM chondrites being commonly forsteritic which could mask secondary forsterite’s presence, a combination of techniques (e.g., XRD and transmission electron microscopy) would likely be required to establish both the presence of secondary olivine and its forsteritic composition.

The results of this study in regard to calcite and its decomposition products vary greatly (Table 1); however, the formation of oldhamite requires either high calcite decomposition temperatures (≥ 610 °C) to allow calcium from calcite to react with sulfur from pentlandite, or rapid heating such that calcite and tochilinite decomposes simultaneously, thus allowing their breakdown products to react. A meteorite with oldhamite that has other heating effects consistent with only moderate degrees of heating (e.g., no secondary olivine, ≤ 600 °C) would be indicative of it having experienced rapid heating on its parent body. However, due to the high susceptibility of oldhamite to terrestrial alteration (Haberle and Garvie 2017), such evidence would require the meteorite be retrieved quickly after its fall.

## Conclusions

Phase transitions that are a result of post-hydration heating experienced by CM chondrites were recreated through the experimental heating of ALH 83100 and Murchison, and investigated with XRD. The 25 °C temperature steps used have allowed for the various mineral reactions to be studied in detail, especially those at lower temperatures (e.g., < 500 °C). Tochilinite decomposition is likely a two-stage process that can begin at 200 °C but requires heating to ≥ 210 °C to complete. Serpentine decomposition starts at 300 °C regardless of the meteorite’s initial composition; however, its decomposition rate may vary depending on the original makeup of the meteorite (e.g., mineralogy and chemical composition), with more aqueously altered meteorites with coarser serpentine grain sizes and more lizardite relative to cronstedtite decomposing at a slower rate. The products of serpentine decomposition are unaffected by these differing rates with transitional structures dehydroxylate I and II appearing at ~ 525 °C and ~ 575–600 °C, respectively, followed by secondary silicates (e.g., forsterite, fayalite, and enstatite). Forsteritic olivine formed faster than fayalitic olivine. Enstatite crystallization requires a temperature of at least 750 °C. Serpentine decomposition rates vary depending on grain size and chemical composition; however, as the initial decomposition temperature is unaffected, this difference is negligible in meteorites heated for more than a few hours.

Calcite decomposition is strongly influenced by its starting morphology, composition, strain, and other factors. Calcite in meteorites that have experienced shock metamorphism will likely decompose at lower temperatures than unshocked meteorites. Less aqueously altered meteorites also may experience calcite decomposition at lower temperatures (e.g., 575 °C) than more altered meteorites due to differences in chemical compositional; however, more studies are needed to test this conclusion. Clinopyroxene is produced from calcite decomposition regardless of the temperature at which it occurs; however, oldhamite formation requires either high temperatures (e.g., ≥ 610 °C) or rapid heating (e.g., reaching peak temperatures in minutes).

For the most part, the starting petrography of a CM chondrite has little impact on the phase transitions that occur due to post-hydration heating. The only major differences observed here pertain to the decomposition of calcite, which can vary greatly. Temperature-timescales have the largest influence on the post-hydration heating effects observed. The mineral transitions observed in this study can be applied to naturally heated meteorites to understand the temperatures they experienced on their parent asteroids, though heating duration must be accounted for.

## Supplementary Information

Supplementary Material 1.

## Data Availability

The data sets used and/or analyzed during the current study are available from the corresponding author on reasonable request.

## Abbreviations

ALH 83100: Allan Hills 83100
ANSMET: Antarctic Search for Meteorites
CM chondrite: ‘Mighei-like’ carbonaceous chondrite
COD: Crystallography Open Database
NASA: National Aeronautics and Space Administration
XRD: X-ray diffraction
