# Supplementary material for: A high-resolution in situ X-ray diffraction study of mineral transitions due to post-hydration heating in CM chondrite meteorites
Source: Earth Planets Space. 2024 Dec 26;76(1):172. doi: 10.1186/s40623-024-02116-2 (PMC11669613; doi:10.1186/s40623-024-02116-2)
Supplement: Supplementary file 1 — Supplementary Material 1. [file 40623_2024_2116_MOESM1_ESM.docx]

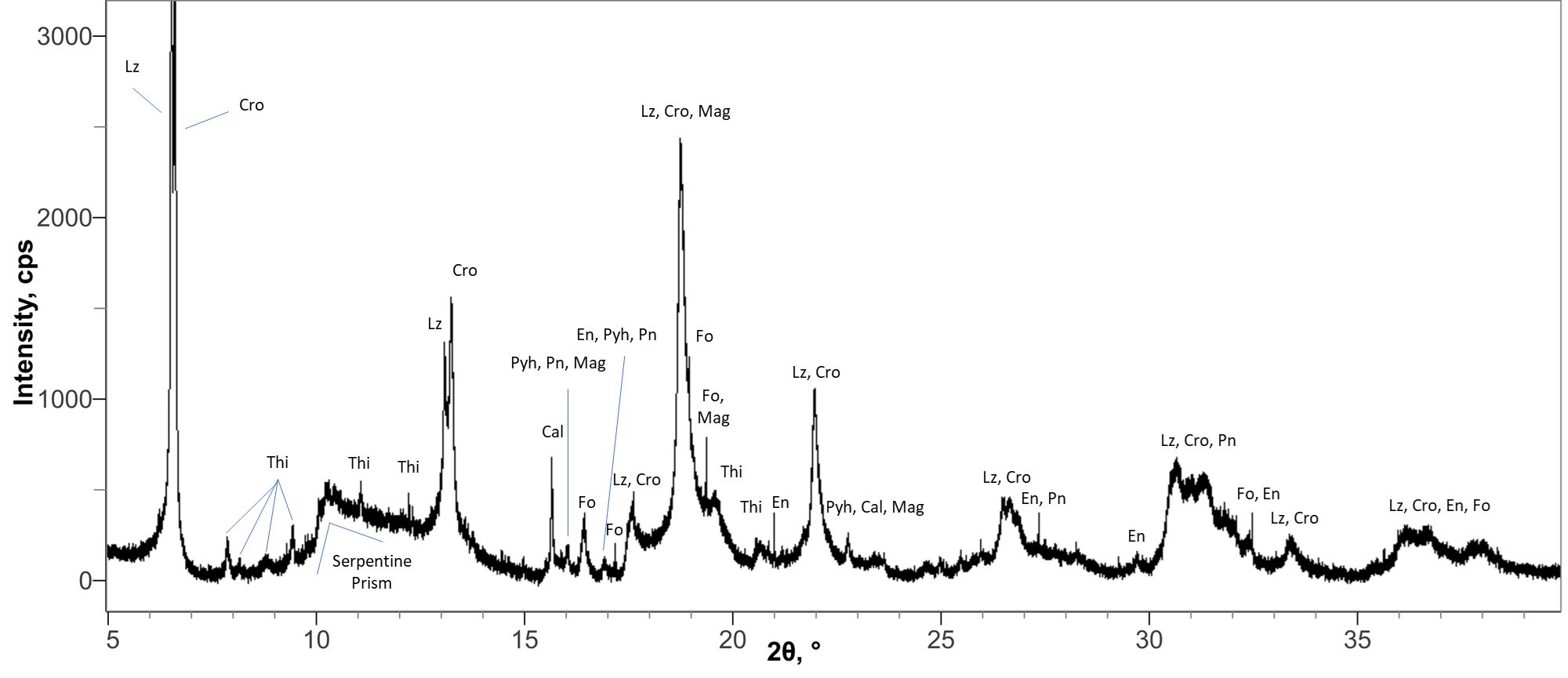


Fig. S1. XRD pattern taken from ALH 83100 at room temperature prior to heating at 0.826890 Å. Phases labelled are lizardite (Lz), cronstedtite (Cro), tochilinite (Thi), pyrrhotite (Pyh), calcite (Cal), magnetite (Mag), forsterite (Fo), enstatite (En), and pentlandite (Pn).


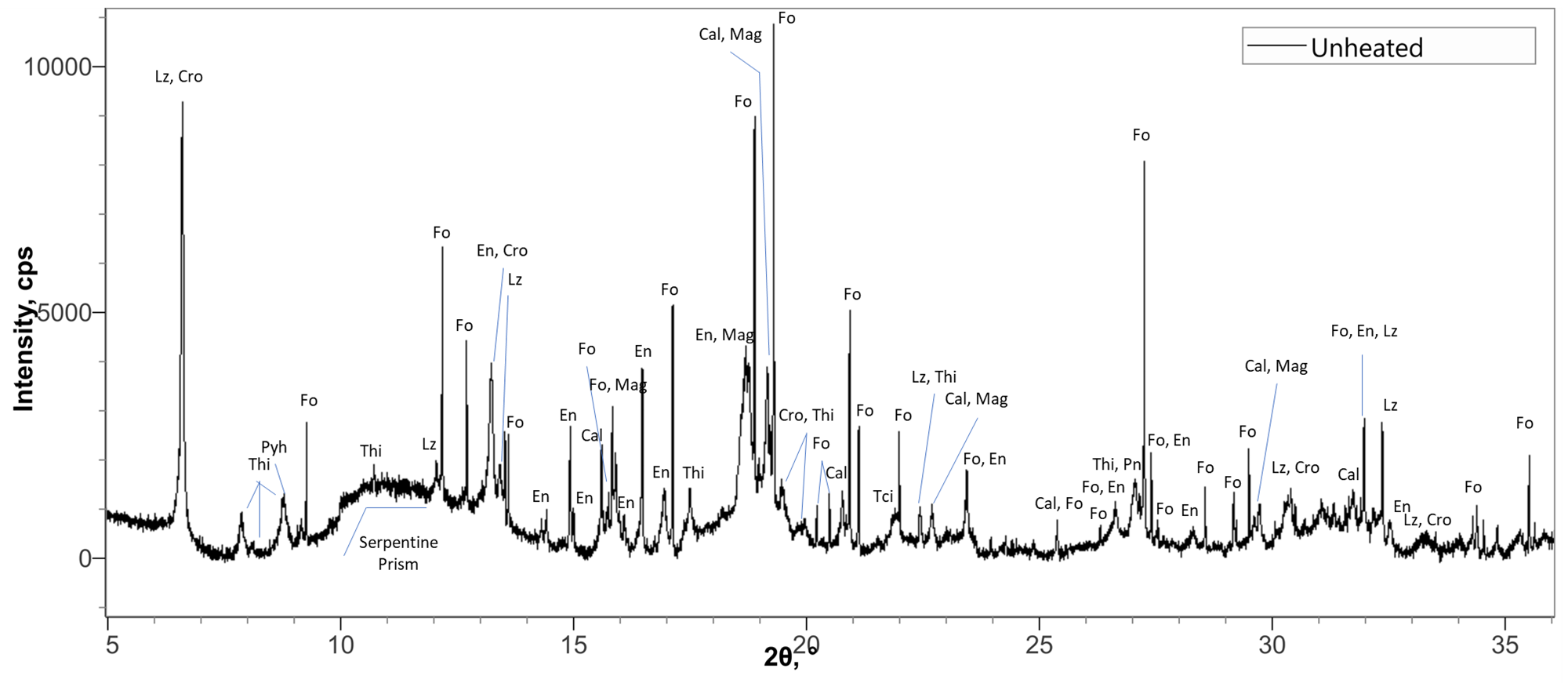


Fig. S2. XRD pattern taken from Murchison at room temperature prior to heating at 0.823845 Å. Phases labelled are lizardite (Lz), cronstedtite (Cro), tochilinite (Thi), pyrrhotite (Pyh), calcite (Cal), magnetite (Mag), forsterite (Fo), enstatite (En), and pentlandite (Pn).


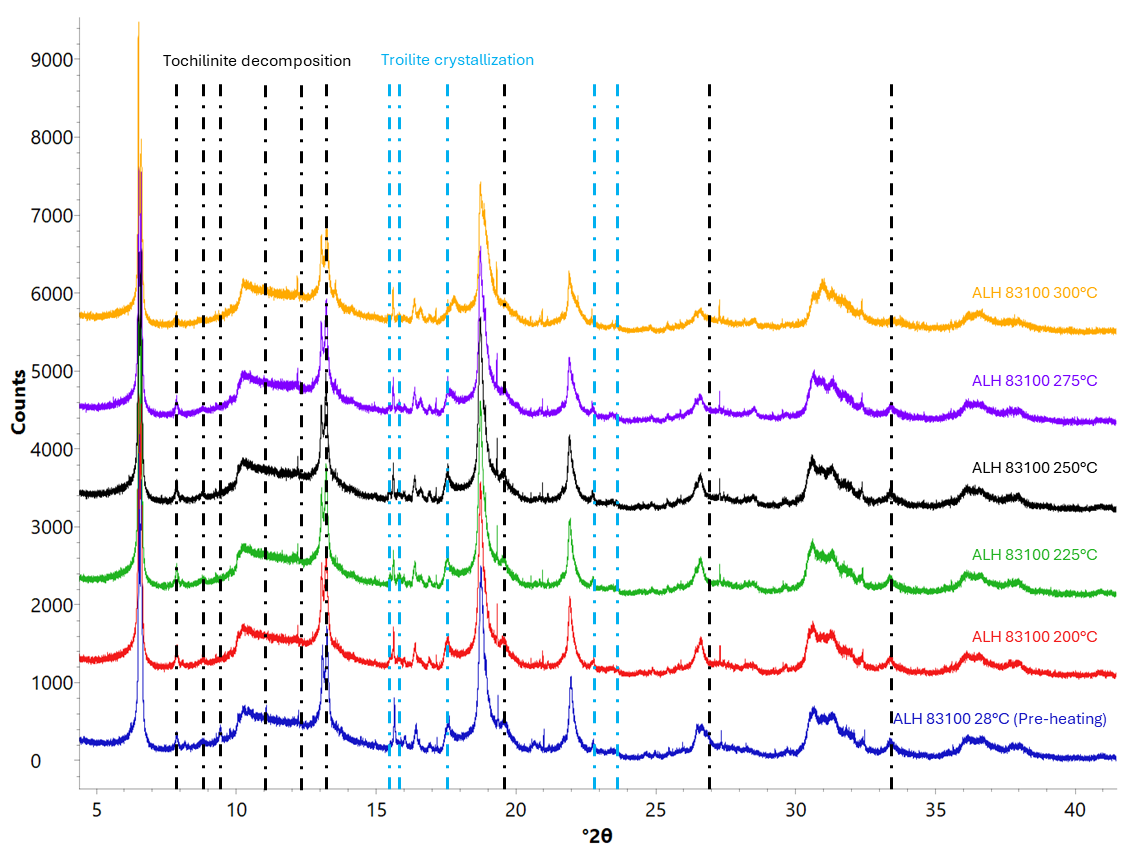


Fig. S3. XRD patterns for ALH 83100 from room temperature to 300°C. Major peak changes relating to incipient tochilinite decomposition, partially beginning at 200°C and accelerating at 275°, and incipient troilite crystallization at 275°C are marked.


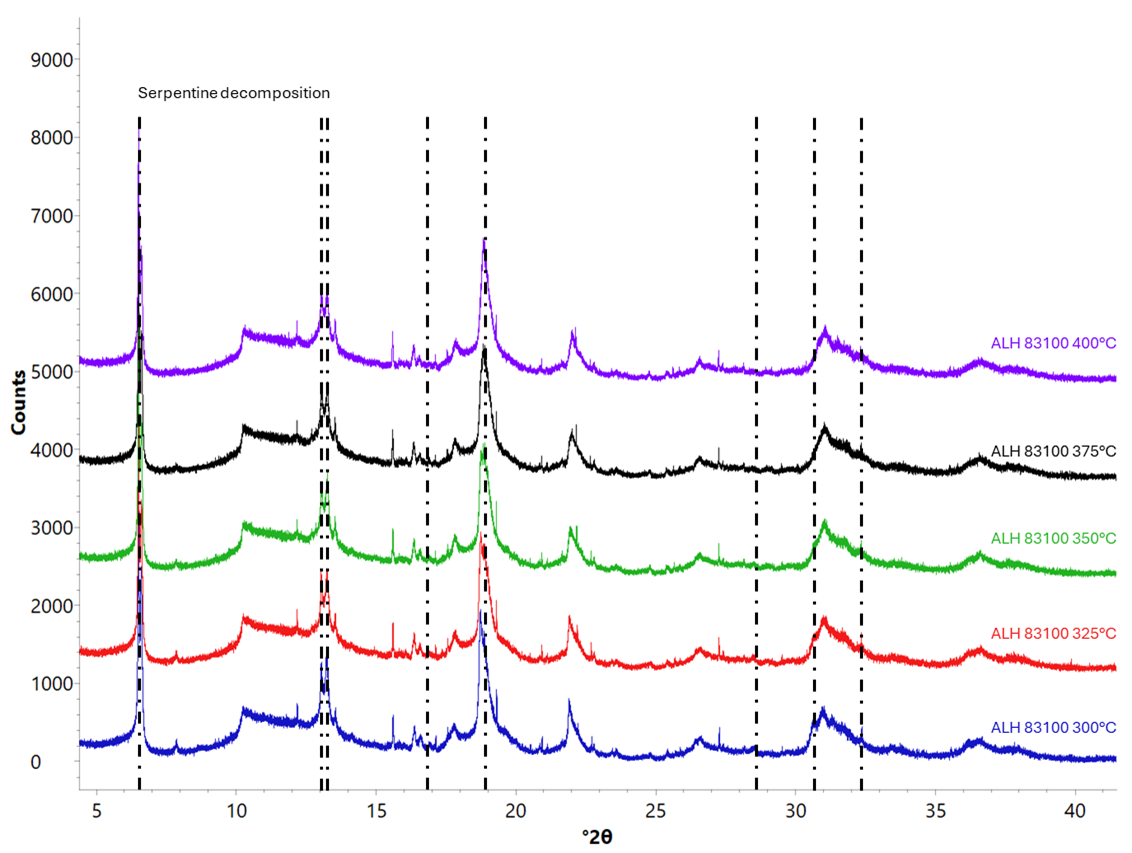


Fig. S4. XRD patterns for ALH 83100 from 300°C to 400°C. Major peak changes relating to incipient serpentine decomposition at 300°C are marked.


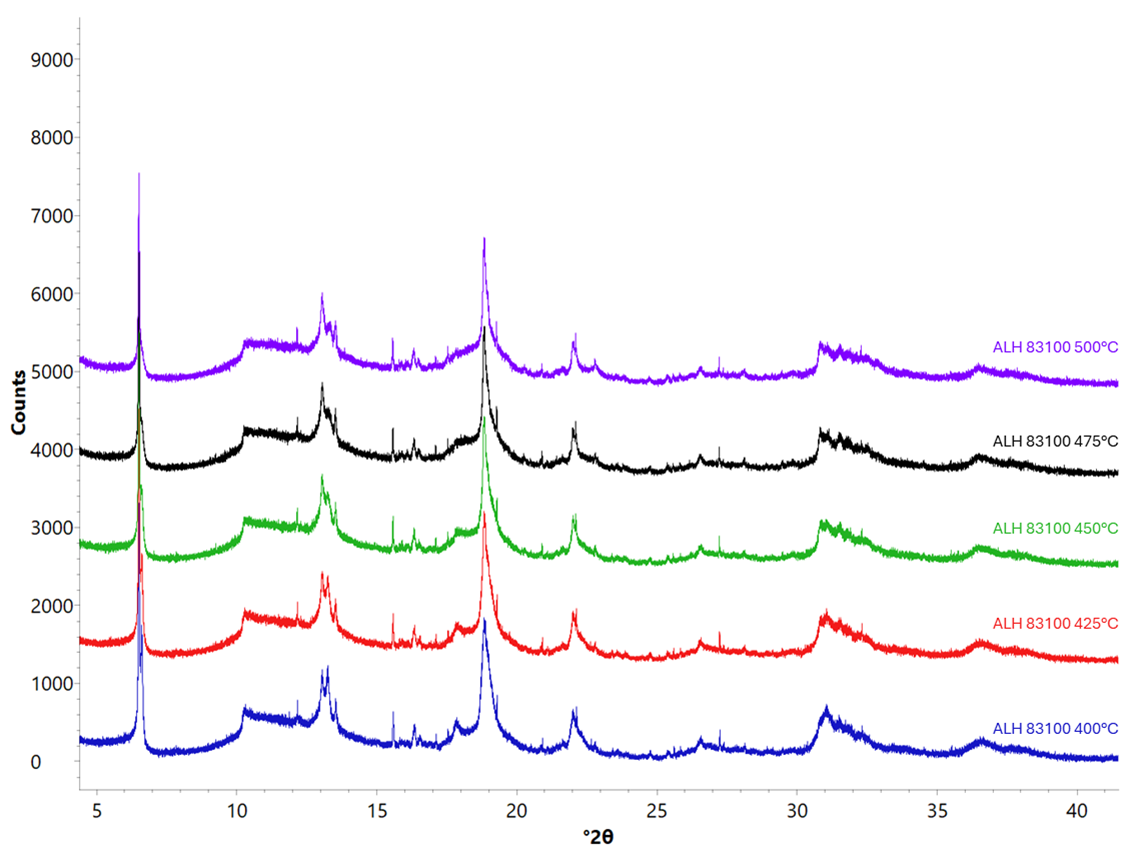


Fig. S5. XRD patterns for ALH 83100 from 400°C to 500°C.


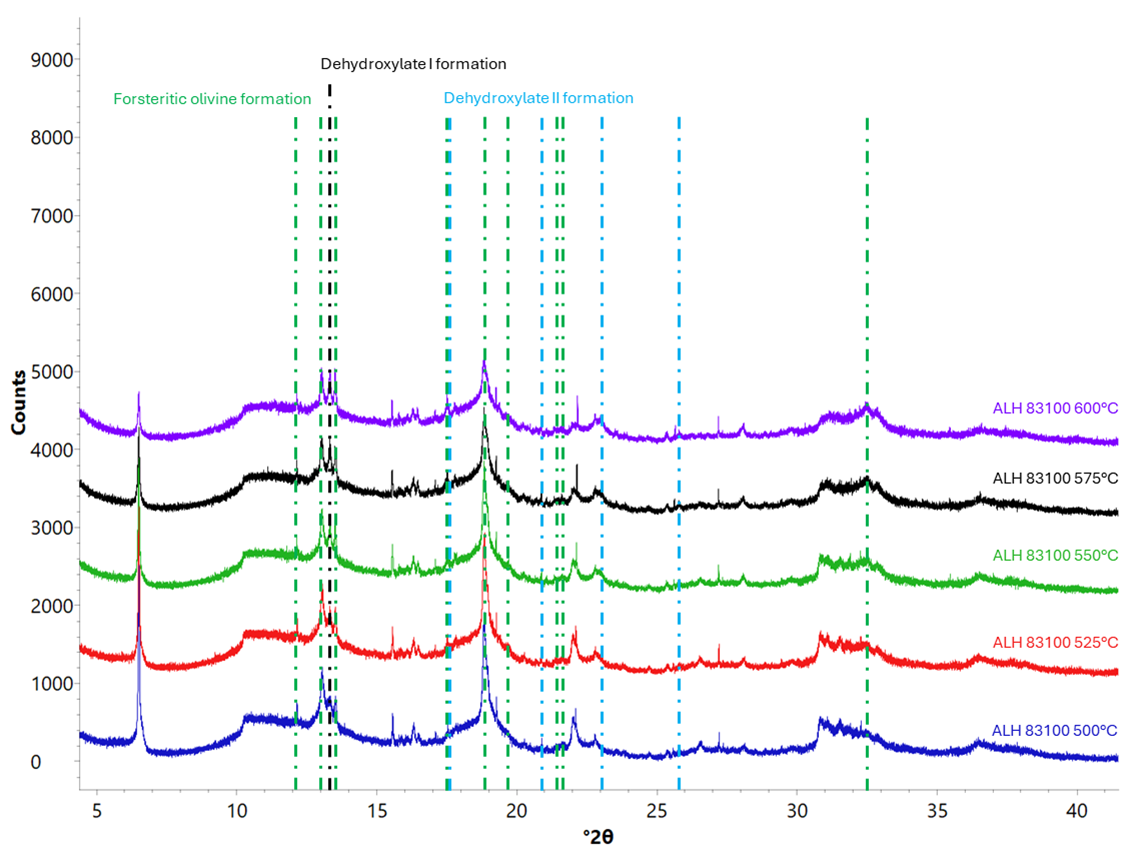


Fig. S6. XRD patterns for ALH 83100 from 500°C to 600°C. Major peak changes relating to incipient dehydroxylate I formation at 525°C, dehydroxylate II formation at 575°C, and forsteritic olivine formation at 600°C are marked.


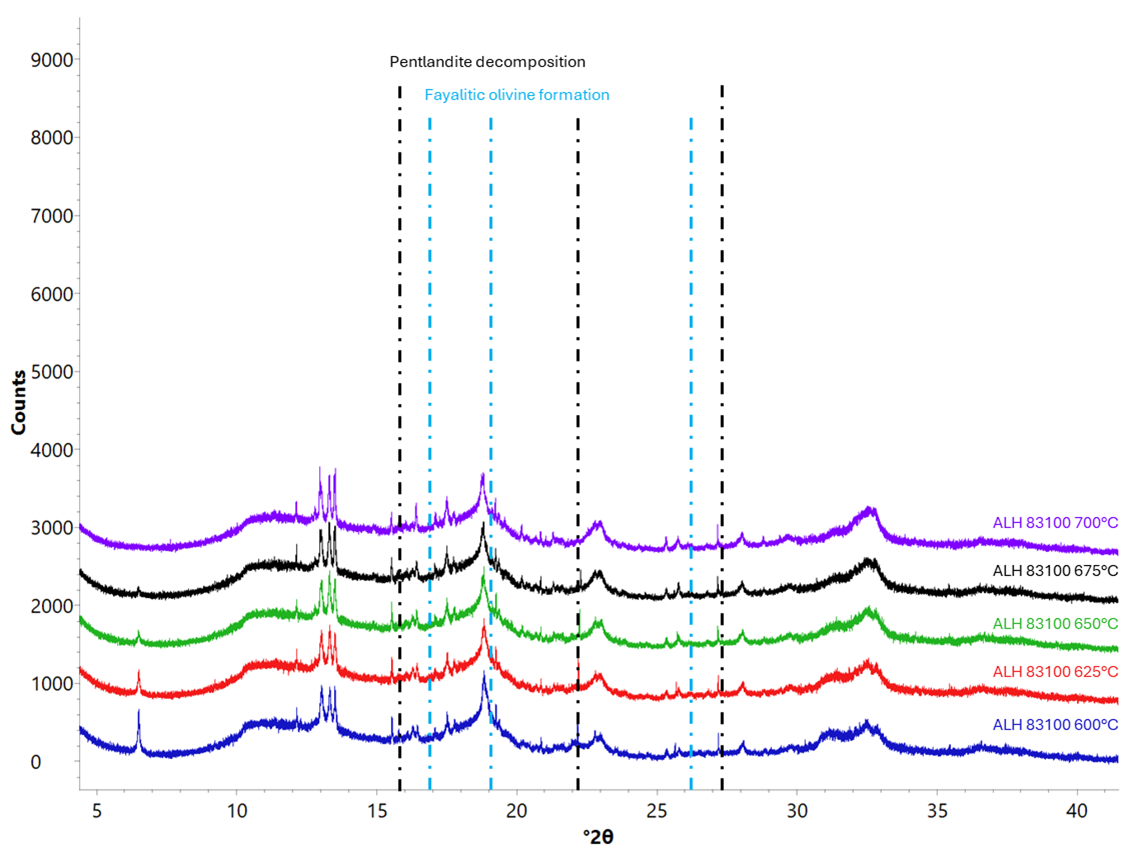


Fig. S7. XRD patterns for ALH 83100 from 600°C to 700°C. Major peak changes relating to incipient pentlandite decomposition at 675°C and fayalitic olivine formation at 650°C are marked.


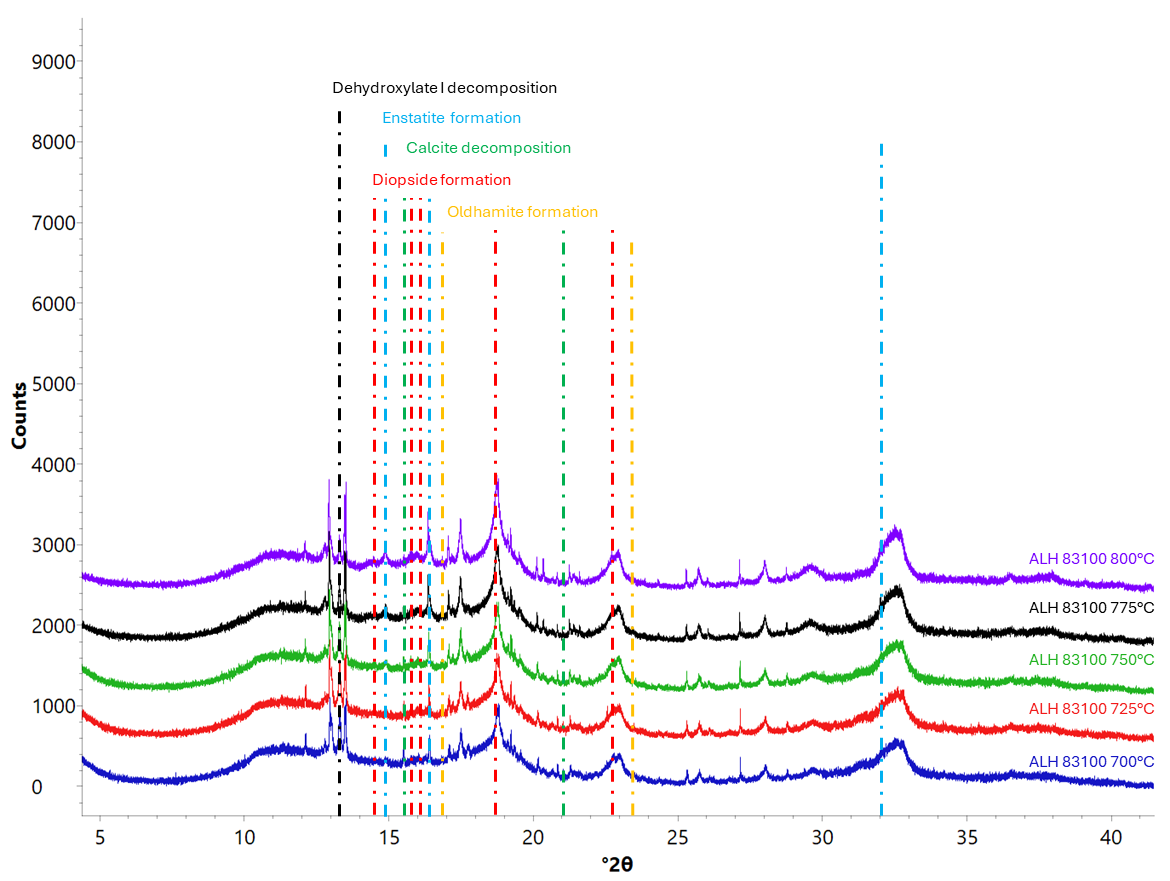


Fig. S8. XRD patterns for ALH 83100 from 700°C to 800°C. Major peak changes relating to incipient dehydroxylate I decomposition at 750°C, enstatite formation at 750°, calcite decomposition at 725°C, diopside formation at 725°C, and oldhamite formation at 725°C are marked.


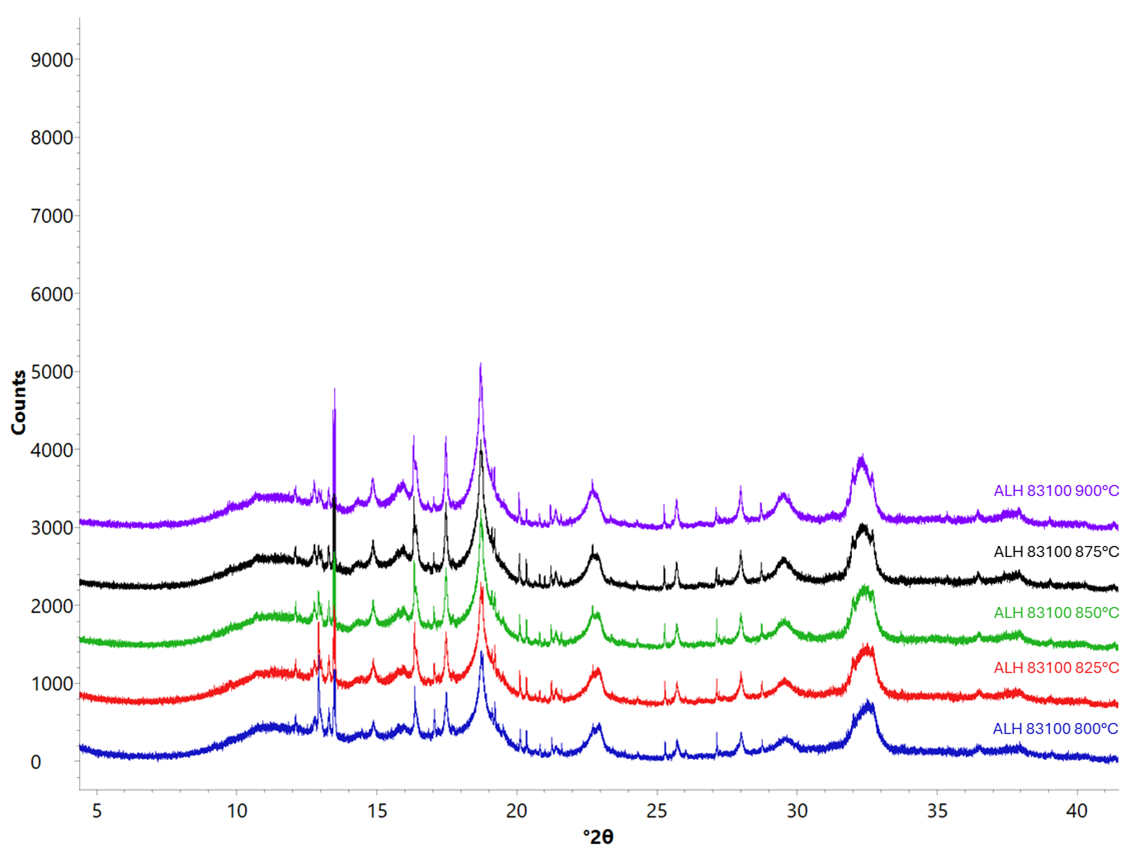


Fig. S9. XRD patterns for ALH 83100 from 800°C to 900°C.


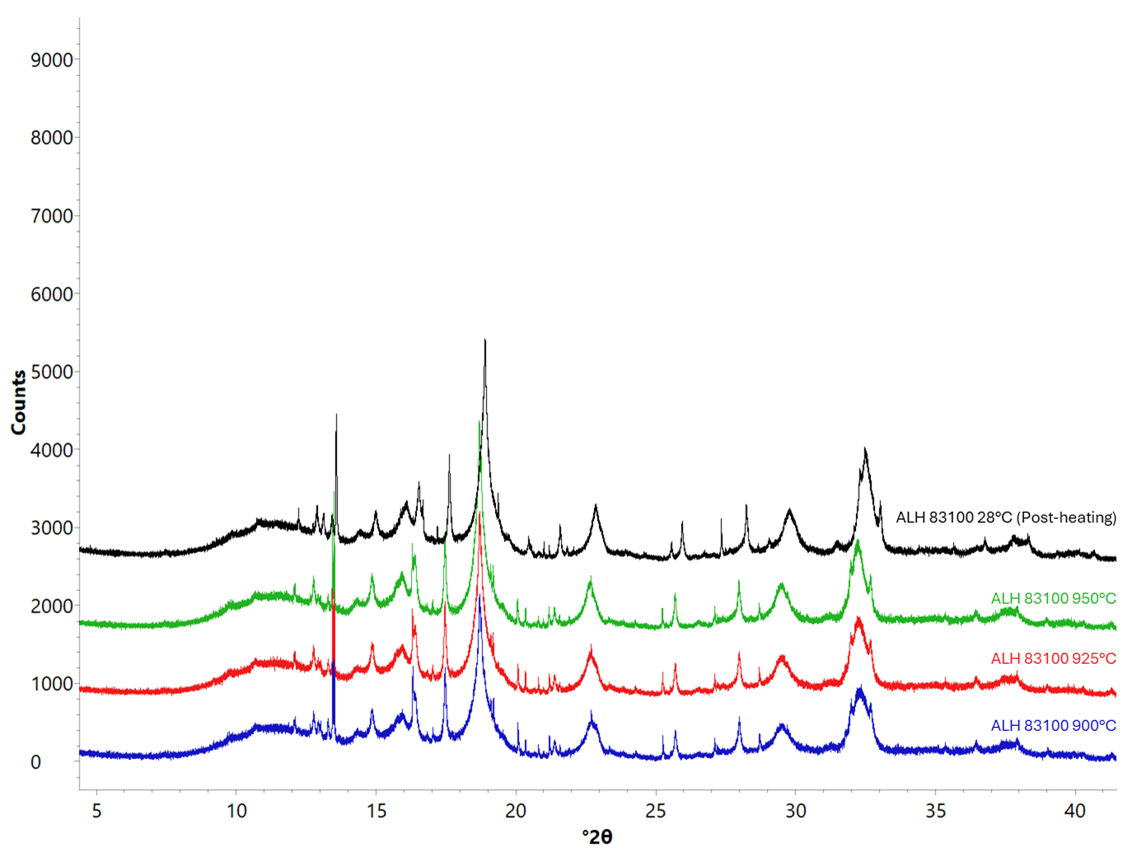


Fig. S10. XRD patterns for ALH 83100 from 900°C to 950°C, as well as an XRD pattern for ALH 83100 at room temperature after heating.


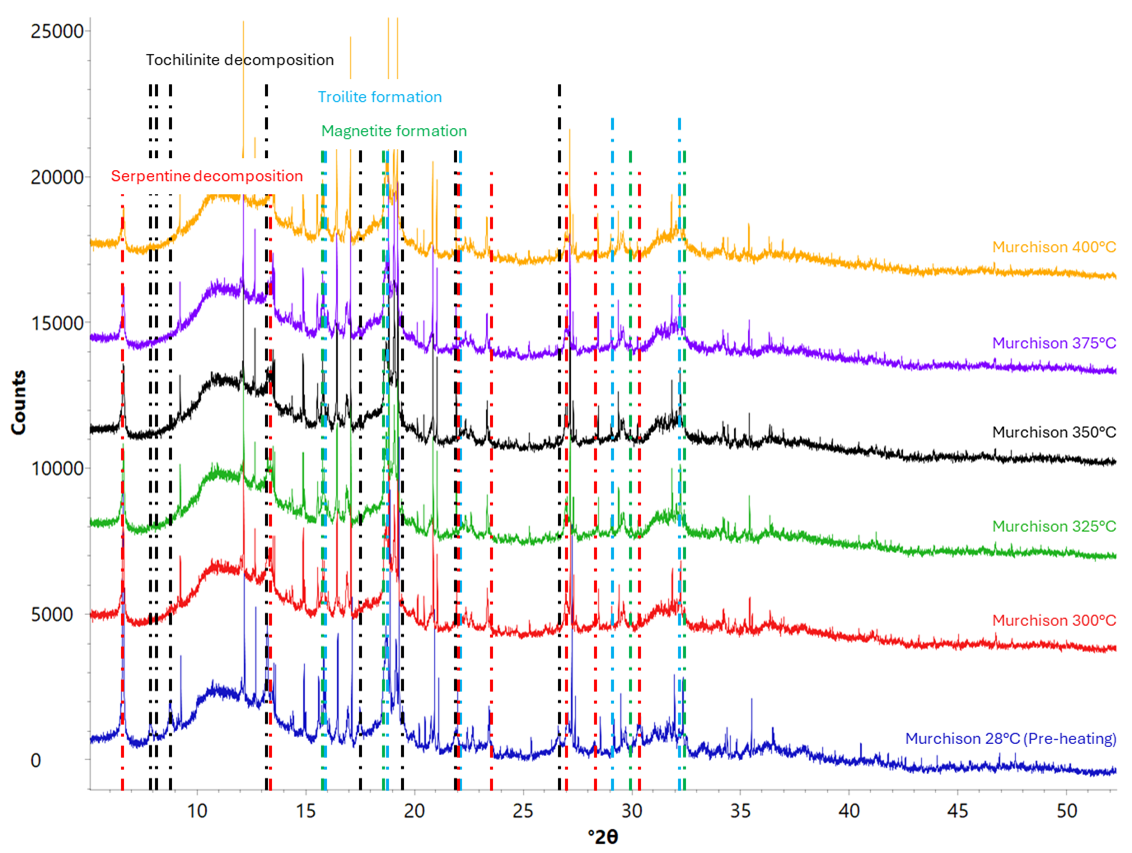


Fig. S11. XRD patterns for Murchison from room temperature to 400°C. Major peak changes relating to tochilinite decomposition at 300°C, troilite formation at 300°C, magnetite formation at 350°C, and serpentine decomposition at 300°C are marked.


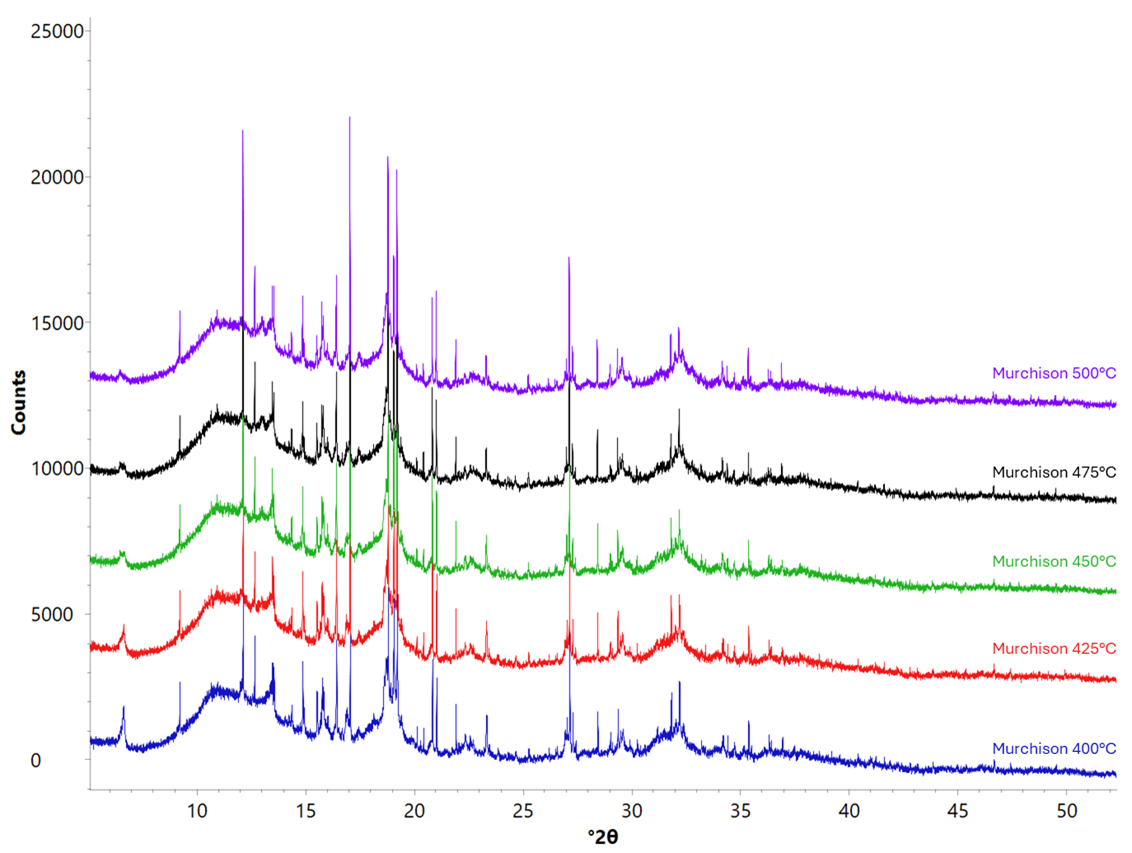


Fig. S12. XRD patterns for Murchison from 400°C to 500°C.


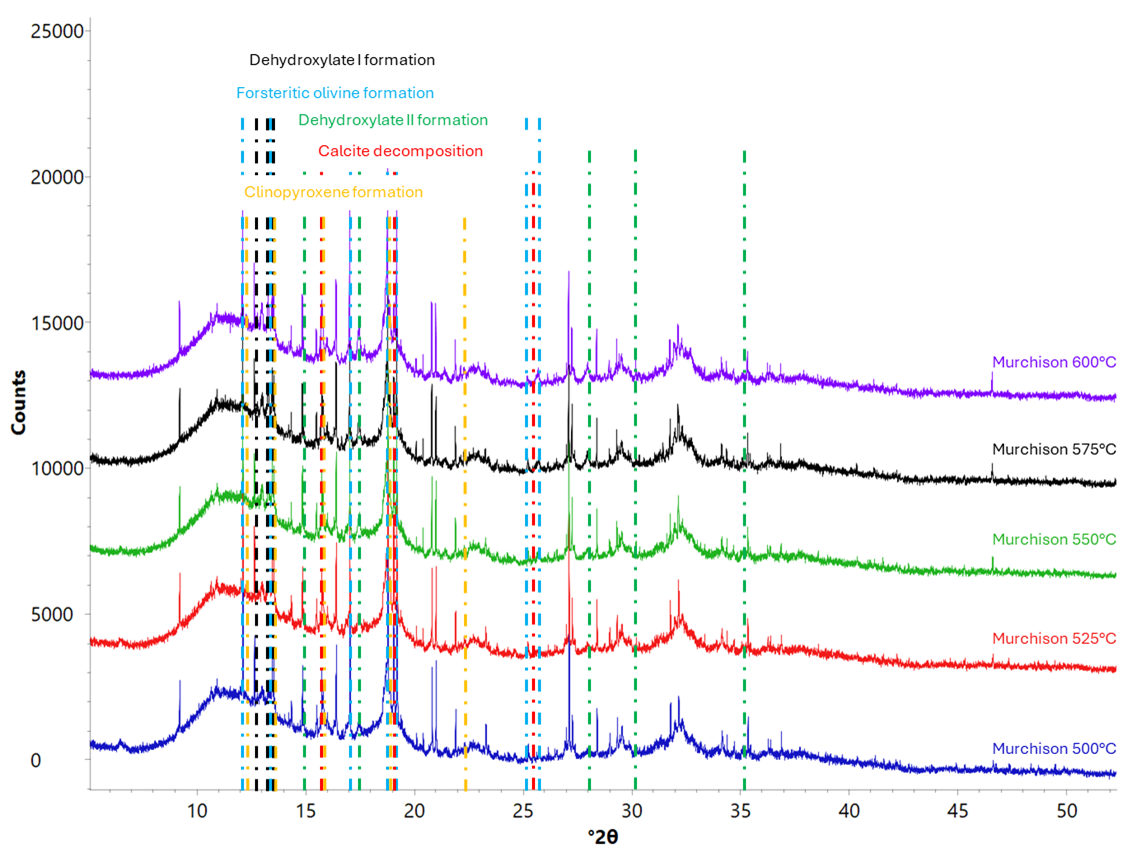


Fig. S13. XRD patterns for Murchison from 500°C to 600°C. Major peak changes relating to incipient dehydroxylate I formation at 525°C, forsteritic olivine formation at 600°C, dehydroxylate II formation at 600°C, calcite decomposition at 575°C, and clinopyroxene formation at 575°C are marked.


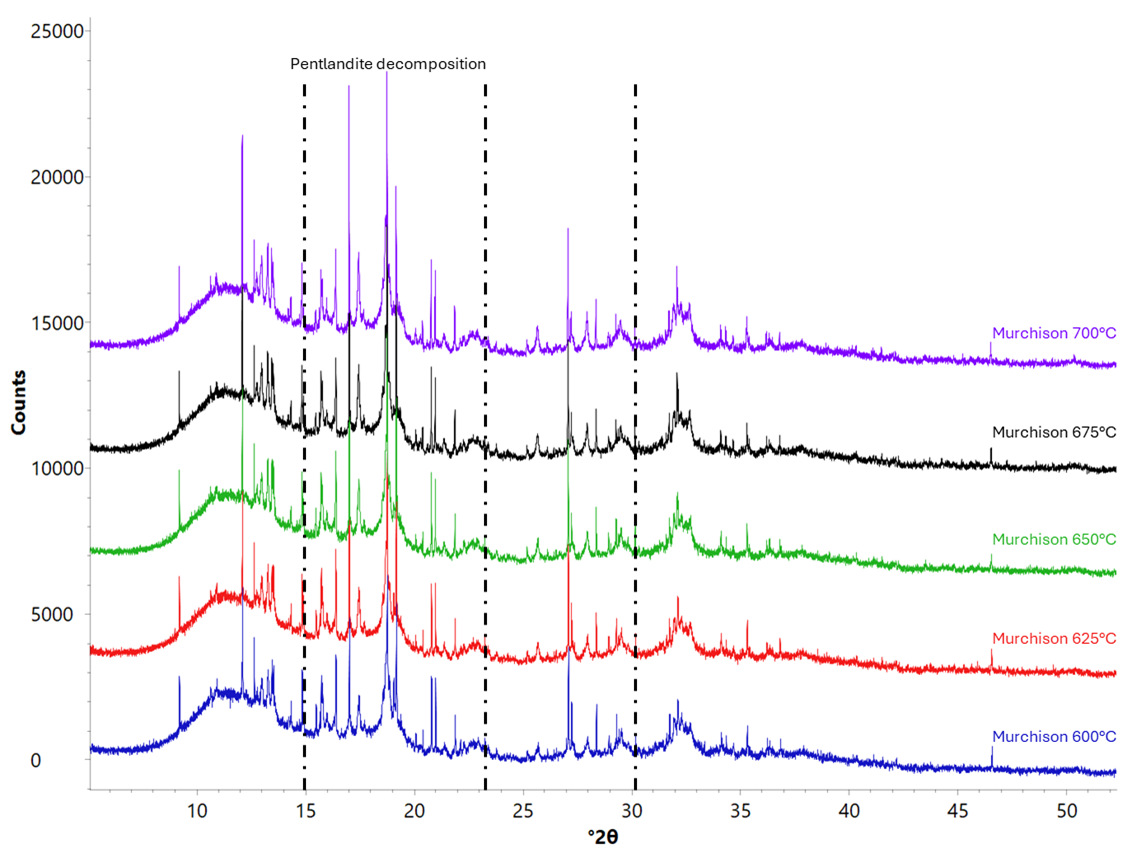


Fig. S14. XRD patterns for Murchison from 600°C to 700°C. Major peak changes relating to incipient pentlandite decomposition at 675°C are marked.


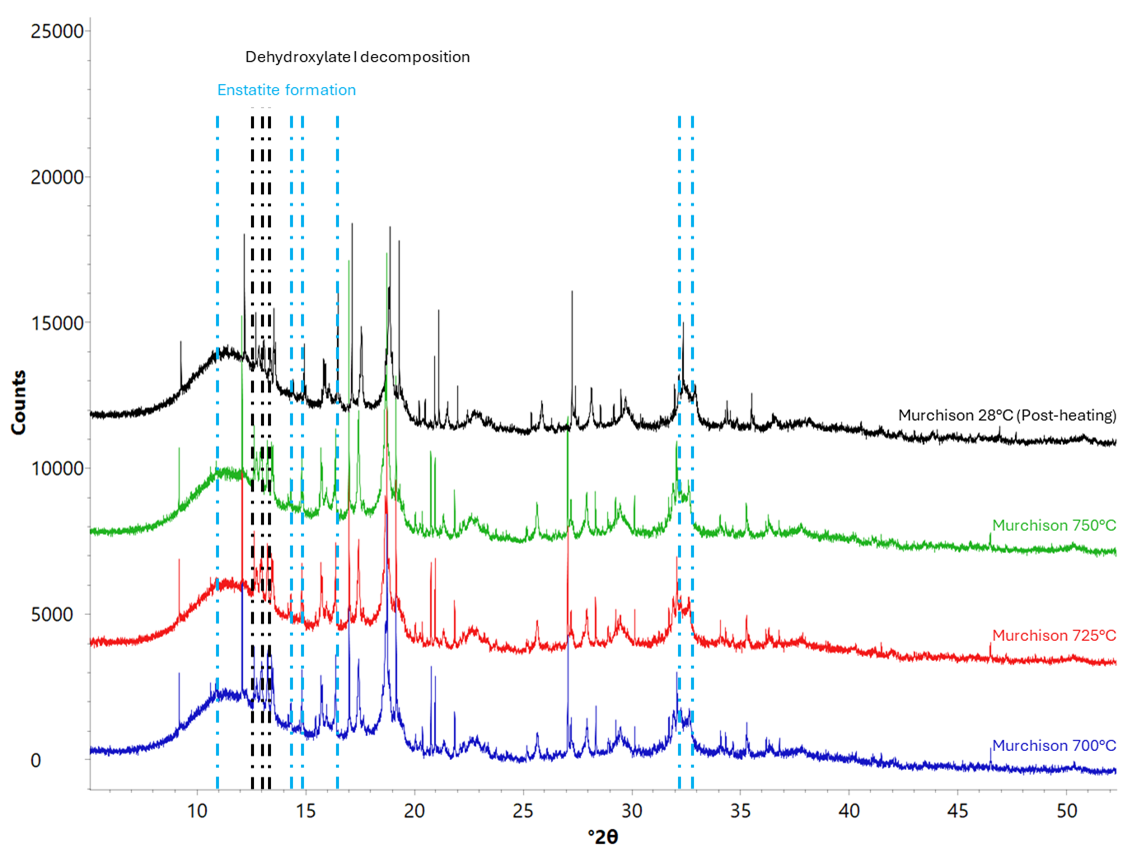


Fig. S15. XRD patterns for Murchison from 700°C to 750°C as well as a room temperature XRD pattern of Murchison taken after heating. Major peak changes relating to incipient dehydroxylate I decomposition occurring during the cooldown to room temperature and enstatite formation occurring at 750°C are marked.
